# Genome-wide association study for biomarker identification of Rapamycin and Everolimus using a lymphoblastoid cell line system

**DOI:** 10.3389/fgene.2013.00166

**Published:** 2013-08-30

**Authors:** Jing Jiang, Brooke L. Fridley, Qiping Feng, Ryan P. Abo, Abra Brisbin, Anthony Batzler, Gregory Jenkins, Pamela A. Long, Liewei Wang

**Affiliations:** ^1^Department of Molecular Pharmacology and Experimental Therapeutics, Mayo ClinicRochester, MN, USA; ^2^Department of Health Sciences Research, Mayo ClinicRochester, MN, USA

**Keywords:** mTOR, pharmacogenomics, genome-wide association, Rapamycin, Everolimus

## Abstract

The mammalian target of rapamycin (mTOR) inhibitors, a set of promising potential anti-cancer agents, has shown response variability among individuals. This study aimed to identify novel biomarkers and mechanisms that might influence the response to Rapamycin and Everolimus. Genome-wide association (GWA) analyses involving single nucleotide polymorphisms (SNPs), mRNA, and microRNAs microarray data were assessed for association with area under the cytotoxicity dose response curve (AUC) of two mTOR inhibitors in 272 human lymphoblastoid cell lines (LCLs). Integrated analysis among SNPs, expression data, microRNA data and AUC values were also performed to help select candidate genes for further functional characterization. Functional validation of candidate genes using siRNA screening in multiple cell lines followed by MTS assays for the two mTOR inhibitors were performed. We found that 16 expression probe sets (genes) that overlapped between the two drugs were associated with AUC values of two mTOR inhibitors. One hundred and twenty seven and one hundred SNPs had *P* < 10^−4^, while 8 and 10 SNPs had *P* < 10^−5^ with Rapamycin and Everolimus AUC, respectively. Functional studies indicated that 13 genes significantly altered cell sensitivity to either one or both drugs in at least one cell line. Additionally, one microRNA, miR-10a, was significantly associated with AUC values for both drugs and was shown to repress expression of genes that were associated with AUC and desensitize cells to both drugs. In summary, this study identified genes and a microRNA that might contribute to response to mTOR inhibitors.

## Introduction

The mammalian target of rapamycin (mTOR), a kinase acting downstream of the PI3K/AKT signaling pathway, is a critical regulator of basic cellular functions and plays an important role in tumor progression. Activated mTOR as a response to nutritional status promotes cell growth, proliferation, motility, and metabolism (Guertin and Sabatini, 2005; Petroulakis et al., 2006) through the regulation of a wide range of cellular activities, including translation, transcription, mRNA turnover, protein stability, actin cytoskeletal organization, and autophagy (Jacinto and Hall, 2003; Inoki et al., 2005). The best characterized function of mTOR in mammalian cells is regulation of protein translation through key downstream effectors of mTOR complex 1 (TORC1), the ribosomal S6 kinase (S6K) and eukaryote initiation factor 4E binding protein (4EBP1). S6K is the major ribosomal protein S6 kinase in mammalian cells. Phosphorylation of the S6 protein by S6K selectively increases the translation of mRNAs containing a tract of pyrimidines motif, which encode ribosomal proteins and other translation regulators, thereby enhancing the overall translation capacity of the cells (Meyuhas, 2000; Inoki et al., 2005). 4EBP1 acts as a translational repressor by binding and inhibiting the eukaryotic translation initiation factor 4E (elF4E), which recognizes the 5′-end cap of eukaryotic mRNAs (Cho et al., 2005; Richter and Sonenberg, 2005). Phosphorylation of 4EBP1 by mTOR results in the dissociation of 4EBP1 from elF4E, thereby relieving the inhibition of elF4E-dependent translation initiation by 4EBP1.

Since aberrant activity of the PI3K/AKT/mTOR pathway is commonly observed in cancer, mTOR inhibitors (e.g., Everolimus, Deferolimus, and Temsirolimus) have emerged as promising therapeutic agents for the treatment of a variety types of cancer, including renal-cell carcinoma, breast carcinoma, non-small-cell lung carcinoma, endometrial carcinoma, glioblastoma, and mantle cell lymphoma (Chapman and Perry, 2004; Rowinsky, 2004; Vignot et al., 2005; Hartford and Ratain, 2007). However, mTOR inhibitors have severe adverse effects such as nephrotoxicity and potential immune suppression (i.e., skin reactions, mucositis, and myelosuppression) (Rowinsky, 2004; Guertin and Sabatini, 2005; Vignot et al., 2005). Many factors contribute to mTOR drug response, with genetic variation being one major factor. To maximize the efficacy and safety of mTOR inhibitors, there is a critical need to identify genetic biomarkers for response and to elucidate specific mechanisms by which these biomarkers might be involved in response to mTOR inhibitors.

In the present study, we aimed to identify novel pharmacogenomic candidates that might contribute to variation in response to two mTOR inhibitors, Rapamycin and Everolimus, using a cell line system consisting of 300 human lymphoblastoid cell lines (LCLs) from three ethnic groups. In addition to cytotoxicity represented by the dose response curves (AUCs) for the two mTOR inhibitors, we have also obtained extensive genomic information for these LCLs, including approximately 1.3 million SNPs, 54,613 mRNA expression probe sets and 228 microRNA probe sets. This model system has previously been utilized successfully to identify genetic biomarkers associated with drug response for a variety of anti-cancer agents (Li et al., 2008, 2009; Pei et al., 2009; Niu et al., 2010), as well as to interpret GWAS signals identified during clinical pharmacogenomic studies of aromatase inhibitor induced musculoskeletal adverse events (Ingle et al., 2010). In this study, we performed genome-wide association (GWA) analyses among SNPs, mRNA expression, microRNA expression, and cytotoxicity phenotypes, as measured by AUC values, for two mTOR inhibitors to identify candidate genes or microRNAs that might contribute to variation in response to mTOR inhibitors. We subsequently validated 13 of these candidate genes and one microRNA using siRNA screening followed by MTS and colony formation assays.

## Materials and methods

### Cell lines

300 EVB-transformed LCLs from healthy subjects (sample sets HD100CAU, HD100AA, HD100HCA) were obtained from the Coriell Cell Repository (Camden, NJ, USA), as reported previously (Li et al., 2008; Niu et al., 2010). All of these samples had been anonymized by the National Institute of General Medical Sciences (NIGMS) before deposit, and all subjects had provided written consent for the use for their DNA and cells for experimental purpose. Two hundred and seventy two LCLs from 87 Caucasian–American (CA), 91 African–American (AA) and 94 Han Chinese–American (HCA) subjects were included in this study. Human diploid fibroblast IMR-90 cell and human renal carcinoma Caki2 cells were provided by Dr. Andrew H. Limper, and Dr. Haidong Dong, respectively, at the Mayo Clinic. The human glioma U87 cell line was purchased from ATCC (no. HTB-14). LCLs were cultured in RPMI 1640 media (Cellgro, VA) supplemented with 15% FBS (Atlanta Biologicals, GA, USA). Caki2 cells were cultured in RPMI 1640 containing 10% FBS. Both the IMR90 and U87 cell lines were grown in DMEM media (GIBCO, Carlsbad, CA, USA) with 10% FBS.

### Transient transfection and RNA interference

SiRNA duplex for candidate genes and negative control, as well as miR-10a inhibitor, microRNA inhibitor negative control, miR-10a mimic and microRNA mimic negative control were all purchased from Dharmacon Inc. (Lafayette, CO, USA). Cells were reversely transfected with 30 nM of siRNA or microRNA mimic/inhibitors with Lipofectamin™ RNAiMax transfection reagent (Invitrogen, Carlsbad, CA, USA).

### Drug treatment

The mTOR inhibitors, Rapamycin and Everolimus, were purchased from Sigma-Aldrich (St. Louis, MO). Lymphoblastoid cells were seeded in 96 well plates 2 h before drug treatment, whereas IMR90, U87, and Caki2 cells were plated 24 h before drug treatment to allow the cells to adhere. Rapamycin and Everolimus were added to the wells at 8 concentrations ranging from 10^−7^ nM to 1 μM for 72 h before cytotoxicity analysis. For colony formation assay, cells were treated with 0.1, 0.25, 0.5, 0.75, and 1 nM concentration of the two drugs.

### Cytotoxicity assay

Cytotoxicity assays with all cell lines were performed with the CellTiter-96® AQ_ueous_ MTS Proliferation Assay (Promega Corporation, Madison, WI, USA) in 96 well plates. LCLs were seeded at a density of 5 × 10^5^cells/ml (100 μl/well), and IMR90, U87 and Caki2 cells were plated at 2.5 × 10^4^ cells/ml (100 μl/well). Cytotoxicity was measured by adding 20 μ l MTS reagent to each well 72 h after drug treatment. Cytotoxicity for human tumor cell lines and fibroblasts were performed using the same procedure except that in this case, cells were incubated overnight before drug treatment.

### Colony formation assay

Caki2 cells were seeded in 6 well plates. Twenty-four hours later, drugs were added and the cells were incubated for an additional 12 days. Colonies were then fixed with methanol, stained with crystal violet (Bio-Rad Laboratories, Inc. Hercules, CA, USA) and counted visually.

### Real-time quantitative reverse transcription-PCR (qRT-PCR)

mRNA was extracted by the use of ZR RNA MiniPrep™ kit (Zymo Research, Irvine, CA, USA), followed by one-step qRT-PCR performed with the SYBR® Green PCR Master Mix kit (Applied Biosystems Inc., Foster City, CA, USA). microRNA was extracted with the miRNeasy Mini Kit (QIAGEN), reverse transcribed with miScript Reverse Transcription Kit (QIAGEN) and detected by the use of the miScript SYBR Green PCR Kit (QIAGEN). Specific primers for mRNA and microRNA amplifications were purchased from QIAGEN.

### Genome-wide SNP and expression data

The genotyping and expression array data were obtained for all 272 LCLs and were quality controlled as previously described (Li et al., 2008; Niu et al., 2010). These data are publically available from the NCBI Gene Expression Omnibus (http://www.ncbi.nlm.nih.gov/geo) under SuperSeries accession numbers GSE24277 and GSE23120. Briefly, DNA for all of the LCLs were genotyped in the Genotype Shared Resource (GSR) at the Mayo Clinic, using Illumina HumanHap 550K and 510S BeadChips containing 561,298 and 493,750 SNPs, respectively. In addition, we also obtained publically available Affymetrix SNP Array 6.0 Chip SNP data involving 643,600 SNPs for these cells. SNPs with call rate less than 95%, minor allele frequency (MAF) less than 0.05 or Hardy–Weinberg Equilibrium (HWE) *p*-values less than 0.001 were removed. Overall, 1,348,798 SNPs passed quality control. Total RNA was extracted using RNeasy Mini kits (QIAGEN Inc., Valencia, CA, USA). mRNAs were assayed after hybridization to Affymetrix U133 Plus 2.0 GeneChips (54,613 probe sets), as previously described (Li et al., 2008; Niu et al., 2010).

### microRNA expression data

MicroRNA was extracted from each of the cell lines using the mirVana™ microRNA isolation kit (Ambion, Austin, TX, USA). microRNA quality was tested using an Agilent Bioanalyzer, followed by microRNA BeadArray (Illumina, Inc., San Diego, CA, USA), as described previously (Cunningham et al., 2009). Briefly, total RNA were polyadenylated and then reversely transcribed into cDNA using a biotinylated oligo-dT primer with a universal PCR sequence at its 5′-end. This was followed by annealing of the microRNA-specific oligonucleotide (MSO) pool. Common primers were used to amplify the cDNA templates. The single-stranded PCR products were hybridized to the Sentrix Array Matrix (SAM), where the fluorescently labeled strand binds to the bead on the array containing the complementary address sequences. The SAMs were imaged using an Illumina's BeadArray Reader to measure fluorescence intensity, and were analyzed by using Illumina's BeadStudio version 3.1.1. After removing probes with =80% missing (using a *p*-value detection threshold of 0.01) and individual cell lines with =50% missing, 453 probes out of the initial 733 probe sets for 282 individual samples remained. Finally, probes with SD of expression levels among and of the cell lines < 0.40 were removed, leaving 228 probes for analysis.

### Statistical analysis

A detailed description of analysis methods for assessing the association of cytotoxicity phenotypes with SNP and/or mRNA expression data using these LCLs has been described elsewhere (Li et al., 2008, 2009; Niu et al., 2010). Cytotoxicity phenotypes were determined by the best fitting curve using the R package “drc” (dose response curve) (http://cran.r-project.org/web/packages/drc.pdf) based on a logistic model, either 4 parameter logistic, 4 parameter logistic with top = 100%, or 4 parameter logistic with bottom = 0%. The AUC phenotype was determined using the best fitting curve by numerically determining the area under the curve from dose 10^−7^ nM to 1 μM. Since the LCLs represent variation from different sexes and races, the AUC phenotype was Van der Waerden transformed, adjusted for sex, race, and population stratification as previously described (Li et al., 2008; Niu et al., 2010), and standardized for association analysis. SNP data was assessed by population stratigication using the method described by Price et al. (2006). In addition, expression array data was adjusted on standardized residuals for gender, race and batch after Log2 transformation and GCRMA normalization (Ballman et al., 2004; Wu et al., 2004). MicroRNA probes were transformed using a van der Waerden transformation followed by adjusting for all the factors as expression data. Pearson correlations were calculated to quantify the association between adjusted SNPs and AUC values. Similar correlation analyses were also performed between AUC values with normalized and adjusted mRNA expression microRNA data. False discovery rate *Q*-values (Storey, 2003, 2002) were computed for each test. For siRNA and miR-10a transfection experiments, group mean values for AUC and gene expression were compared by using Student's *t*-test.

## Results

### Cytotoxicity of rapamycin and everolimus in lymphoblastoid cell lines

**Figure 1:**
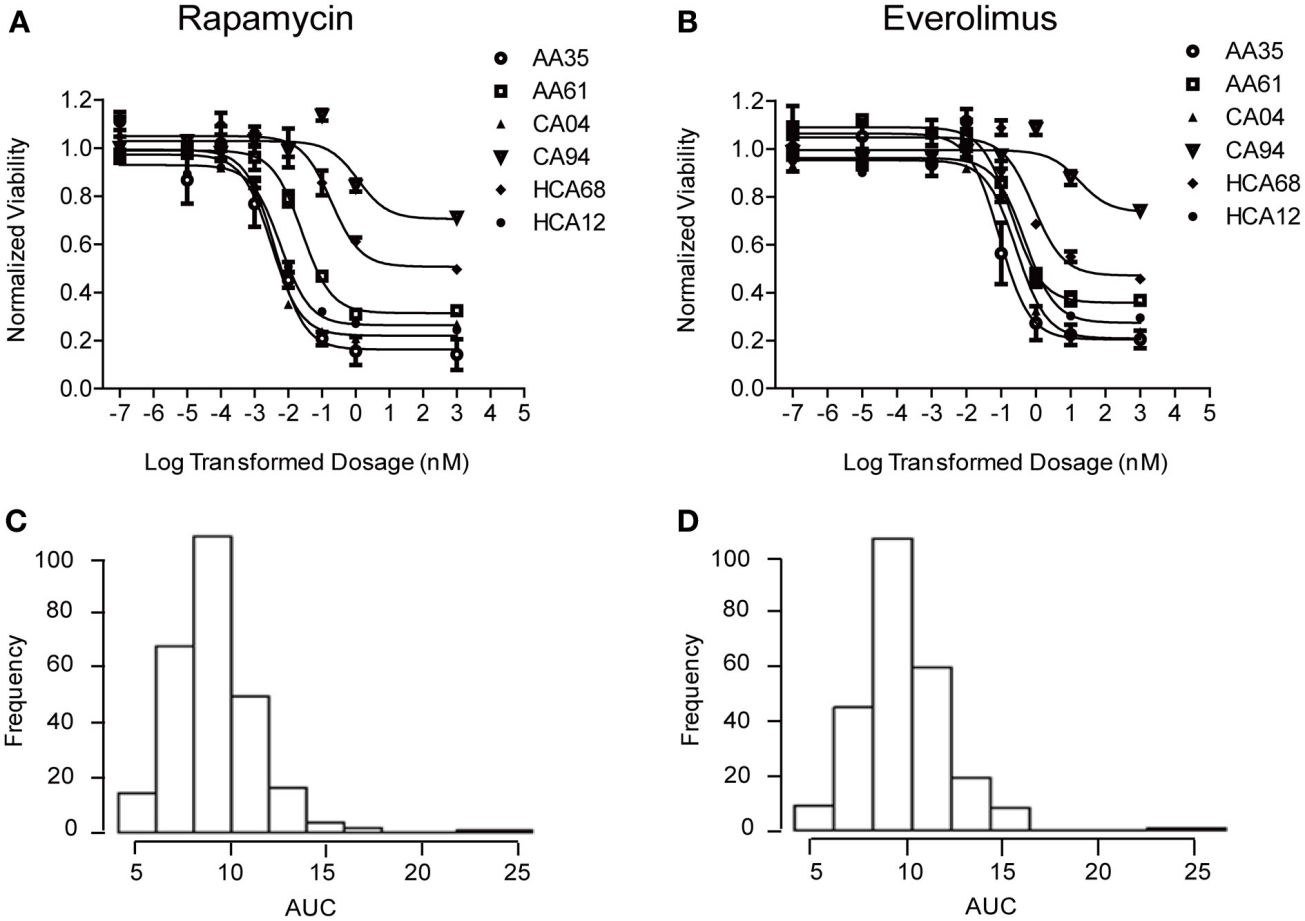
**Cytotoxicity of Rapamycin and Everolimus**. Representative cytotoxicity dose response curves for Rapamycin **(A)** and Everolimus **(B)**. Two cell lines from each of the three ethnic groups studied (AA, African American, CA, Caucasian American and HC, Han Chinese American) were selected to illustrate a range of Rapamycin and Everolimus cytotoxicity. The x-axis indicates the log transformed dosage (nM) and the y-axis indicates the cell viability normalized to control (without drug treatment). Symbols represent individual cell line from different ethnic groups. Histograms of frequency distributions of AUC values for Rapamycin **(C)** and Everolimus **(D)** for 272 lymphoblastoid cell lines.

Cytotoxicity studies were performed to determine the variation of drug response (sensitivity or resistance) to Rapamycin and Everolimus among 272 individual LCLs from three ethnic groups. Representative cytotoxicity data for Rapamycin and Everolimus demonstrated the variation in drug response among individual cell lines (refer to Figures 1A,B). AUC values for each cell line were calculated to capture the entire cytotoxicity curve. The frequency distribution of AUC values for both drugs were shown in Figures 1C,D. The mean AUC values ± standard error (SE) for Rapamycin and Everolimus were 9.2 ± 0.15 and 9.6 ± 0.14, respectively. The AUC values for the two mTOR inhibitors were highly correlated (*R* = 0.833 and *p* = 1.78e−70). Neither race (*P* = 0.458, Rapamycin; *P* = 0.096, Everolimus) nor gender (*P* = 0.252, Rapamycin; *P* = 0.292, Everolimus) was significantly associated with Rapamycin or Everolimus AUC values (Supplementary Figure S1).

### Genome-wide associations for candidate gene identification

#### mRNA expression vs. cytotoxicity

**Figure 2:**
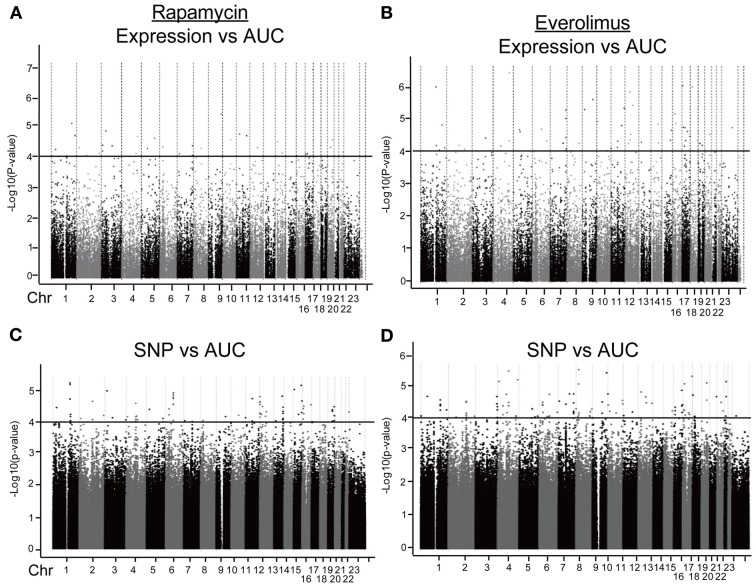
**Genome-wide association of mRNA expression and SNPs with Rapamycin and Everolimus cytotoxicity**. Association of basal gene expression with AUC values for Rapamycin **(A)** and Everolimus **(B)**. Genome-wide association of SNPs with AUC values for Rapamycin **(C)** and Everolimus **(D)**. The x-axis represents chromosomal locations of gene probe sets or SNPs, and the y-axis represents the −log_10_(*P*-value) for the association of individual expression array probe sets or SNPs with AUC values. A *P*-value of 10^−4^ is represented by a horizontal line.

We first identified candidate genes with expression levels that were strongly correlated with cytotoxicity AUCs for Rapamycin and Everolimus, respectively (refer to Figures 2A,B). Only probe set 229939_at (*FLJ35220)* for Rapamycin and 229419_at (*FBXW7*) for Everolimus was genome-wide significant after Bonferroni correction (*P* = 0.006 and 0.02, respectively). Forty-nine probe sets (for 48 genes) and 56 probe sets (for 55 genes) were found to be associated with Rapamycin and Everolimus AUCs with *P* = 10^−4^ (Supplementary Tables S1, S2). Among these probe sets, 16 probe sets (genes) overlapped between the two drugs. Additionally, 3 and 12 genes were highly associated with both Rapamycin and Everolimus AUCs with *P* < 10^−5^, respectively. The most significant probe set for an annotated gene was *PBX3* (*P* = 3.45 × 10^−6^) for Rapamycin and *FBXW7* for (*P* = 3.88 × 10^−7^) for Everolimus. Two genes were found to have 2 probe sets associated with AUC values for each of the drugs (*P* < 10^−4^): *IQSEC1* (203906_at, *P* = 3.70 × 10^−5^; 203907_s_at, *P* = 5.82 × 10^−5^) and *ZNF765* (1558942_at, *P* = 6.84 × 10^−5^; 1558943_x_at, *P* = 3.49 × 10^−5^) for Rapamycin; and *FBXW7* (229419_at, *P* = 3.88 × 10^−7^; 222729_at, *P* = 4.78 × 10^−5^) and *GIMAP1* (1552316_a_at, *P* = 5.48 × 10^−6^; 1552315_at, *P* = 9.63 × 10^−5^) for Everolimus.

**Figure 3:**
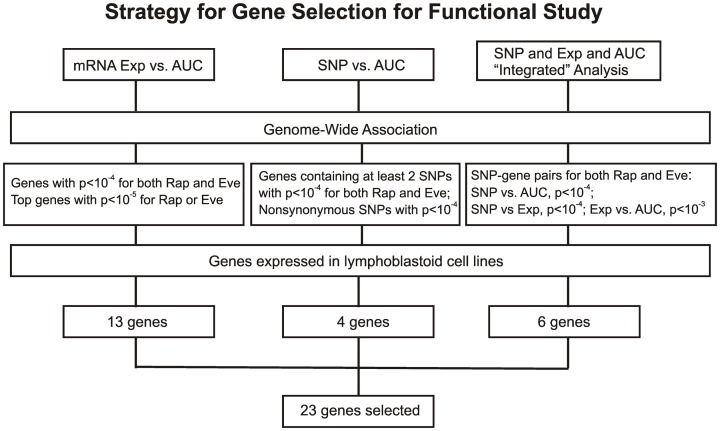
**Schematic diagram of the strategy for selecting candidate genes for functional validation**. A total of 23 candidate genes were selected based on genome-wide associations of expression (Exp) vs. AUC, SNP vs. AUC and an “Integrated” analysis, as described in the text.

For the functional validation, we included the 16 overlapping genes for both drugs with *P* < 10^−4^, genes with *P* < 10^−5^ for Rapamycin or Everolimus, as well as the 4 genes that had 2 probe sets associated with AUC values with *P* < 10^−4^ for each drug. Among those genes, we then removed genes with low expression levels in the LCLs (<50 after GCRMA normalization). Therefore, 13 genes were selected for inclusion in the subsequent functional validation studies (refer to Table 1A and Figure 3).

Table 1**Candidate genes selected for siRNA screening based on GWA analysis**.

**Table:** 

**A. mRNA Exp vs. AUC (Panel 1)**								
**Gene symbol**	**Chr**.	**Probe id**	**Rapamycin**			**Everolimus**		
			***P***	R^*^	Q^**^	***P***	R^*^	Q^**^
BTG2	1	201236_s_at	6.97E-06	0.27	0.10	1.56E-05	0.26	0.05
FBXW7	4	229419_at	1.95E-05	0.26	0.10	3.88E-07	0.30	0.01
STAU1	20	207320_x_at	2.48E-05	0.25	0.10	3.04E-05	0.25	0.05
GIMAP7	7	228071_at	3.91E-05	−0.25	0.10	3.80E-05	−0.25	0.06
PHLDA1	12	217996_at	4.48E-05	0.24	0.10	3.86E-06	0.28	0.03
NDUFAF2	5	228355_s_at	4.75E-05	−0.24	0.10	2.17E-05	−0.25	0.05
SLC39A9	14	222445_at	6.79E-05	0.23	0.10	1.48E-05	0.26	0.05
GIMAP1	7	1552316_a_at	8.16E-05	−0.24	0.10	5.48E-06	−0.27	0.03
ECOP	7	208091_s_at	9.42E-05	0.23	0.10	1.05E-06	0.29	0.01
MGLL	3	225102_at	1.04E-04	0.23	0.10	3.94E-05	0.25	0.06
PBX3	9	204082_at	3.45E-06	0.28	0.08			
ZNF765	19	1558942_at	6.84E-05	0.24	0.10			
		1558943_x_at	3.49E-05	0.25	0.10			
GIMAP6	7	229367_s_at				9.79E-06	−0.2646	0.04

**Table:** 

**B. SNP vs. AUC (Panel 2)**											
**Gene symbol**	**Chr**.	**SNP**	**Position**	**Location**	MAF^***^	**Rapamycin**			**Everolimus**		
						***P***	R^*^	Q^**^	***P***	R^*^	Q^**^
MCTP2	15	rs17664713	93119590	3′-Downstream	0.15	4.70E-06	−0.28	0.96	1.60E-05	−0.27	0.97
		rs17732246	93120790	3′-Downstream	0.10	8.63E-05	−0.25	0.96	4.60E-05	−0.26	0.97
ABCC1	16	rs11075286	15935225	5′-Upstream	0.45	3.93E-05	−0.26	0.96	6.30E-05	−0.25	0.97
		rs4148330	15949269	5′-Upstream	0.50	2.27E-05	−0.26	0.96	9.80E-06	−0.27	0.97
BTNL2	6	rs2076523	32478813	Coding region	0.40	2.77E-05	−0.26	0.96			
PITPNM3	17	rs3809835	6347607	Coding region	0.30	7.73E-05	−0.25	0.96

**Table:** 

**C. Integrated analysis (Panel 3)**																	
**SNP**					**Genes**			**SNP vs. EXP**		**SNP vs. AUC**				**EXP vs. AUC**			
										**Rapamycin**		**Everolimus**		**Rapamycin**		**Everolimus**	
**SNP**	**Chr**.	**Closet gene**	**SNP location**	MAF^***^	**Probe id**	**Chr**.	**Gene symbol**	***P***	R^*^	***P***	R^*^	***P***	R^*^	***P***	R^*^	***P***	R^*^
rs10780752	9	C9orf153	3′-Downstream	0.31	222445_at	14	SLC39A9	2.03E-05	−0.26	4.90E-05	−0.25	3.49E-05	−0.26	6.79E-05	0.24	1.48E-05	0.26
rs7543260	1	JUN	5′-Upstream	0.18	203881_s_at	23	DMD	6.09E-05	−0.24	9.78E-05	−0.24	2.35E-05	−0.26	9.54E-04	0.2	2.98E-04	0.22
rs10870177	9	MAN1B1	Intron	0.17	218470_at	12	YARS2	5.26E-05	0.24	8.04E-05	−0.24	1.99E-05	−0.264	1.72E-04	−0.23	5.87E-05	−0.24
rs4144048	2	GYPC	5′-Upstream	0.05	235790_at	14	LOC100131081	6.37E-05	−0.24	6.20E-05	0.25			0.000355	−0.21		

* *Represents the correlation coefficient R-value for associations*.

** *Represents the false discovery rate Q-value*.

*** *Represents the minor allele frequency (MAF)*.

#### SNP vs. cytotoxicity

Next we performed GWA analysis between SNPs and AUC values for both Rapamycin and Everolimus (refer to Figures 2C,D). Although none of SNPs reached genome-wide significance (*P* < 10^−8^), 127 and 100 SNPs had *P* < 10^−4^, while 8 and 10 SNPs had *P* < 10^−5^ with Rapamycin and Everolimus AUC, respectively (Supplementary Tables S3, S4). Seven genes for Rapamycin and 4 genes for Everolimus contained multiple SNPs with *P* < 10^−4^. Among these genes, *ABCC1* and *MCTP2* were common to both drugs, and those genes were both expressed in the LCLs. Therefore we included these two genes in our functional studies. The majority of the top associated SNPs were located in the non-coding regions of genes, except for 2 non-synonymous SNPs, rs2076523 (*P* = 2.77 × 10^−5^) and rs3809835 (*P* = 7.73 × 10^−5^) both for Rapamycin. These SNPs were located in the coding region of *BTNL2* and *PITPNM3*, respectively. For this reason, these 2 genes were also selected for inclusion in the functional studies of their potential possibility to influence cytotoxicity. A total of 4 genes were selected for functional validation based on SNP vs. cytotoxicity associations, as summarized in Table 1B.

#### Integrated analysis

In order to determine if the SNPs that were associated with cytotoxicity might regulate the expression of genes whose mRNA expression influenced cytotoxicity, “integrated” analyses were performed among SNPs, mRNA gene expression and AUC values for the two mTOR inhibitors studied, as described previously (Storey). Specifically, for the top mTOR associated SNPs (i.e., SNPs with *P* < 10^−4^), we determined their association with gene expression using *P* < 10^−4^ as a cutoff. These SNP-associated genes were then narrowed down to those whose mRNA gene expression probe sets were also associated with mTOR cytotoxicity (*P* < 10^−3^). As the focus of these analyses was exploratory in nature and designed to generate a list of potential candidate genes for functional follow-up, we used less stringent criteria for statistical significance for this selection process.

Twenty SNP-gene pairs for Rapamycin and Everolimus were identified through this integrative analysis (Supplementary Tables S5, S6), with 3 common SNP-gene pairs overlapping between both drugs: rs10780752-*SLC39A9*, rs7543260-*DMD*, and rs10870177-*YARS2*, as listed in Table 1C. The *SLC39A9*, *DMD*, and *YARS2* genes, and genes harboring the SNPs (*C9orf153*, *JUN*, and *MAN1B)* were all included for functional validation. In addition, we also included *GYPC* during our functional study, since *GYPC* was one of the 7 genes containing multiple significant SNPs that were associated with Rapamycin cytotoxicity (*P* < 10^−4^) and the 2 significant SNPs (rs4144048 and rs2219206) located in *GYPC* were also associated with expression levels of the *PIP4K2A* and *LOC100131081* genes (*P* < 10^−4^) for Rapamycin.

#### Functional validation of candidate genes in tumor and primary cell lines

**Figure 4:**
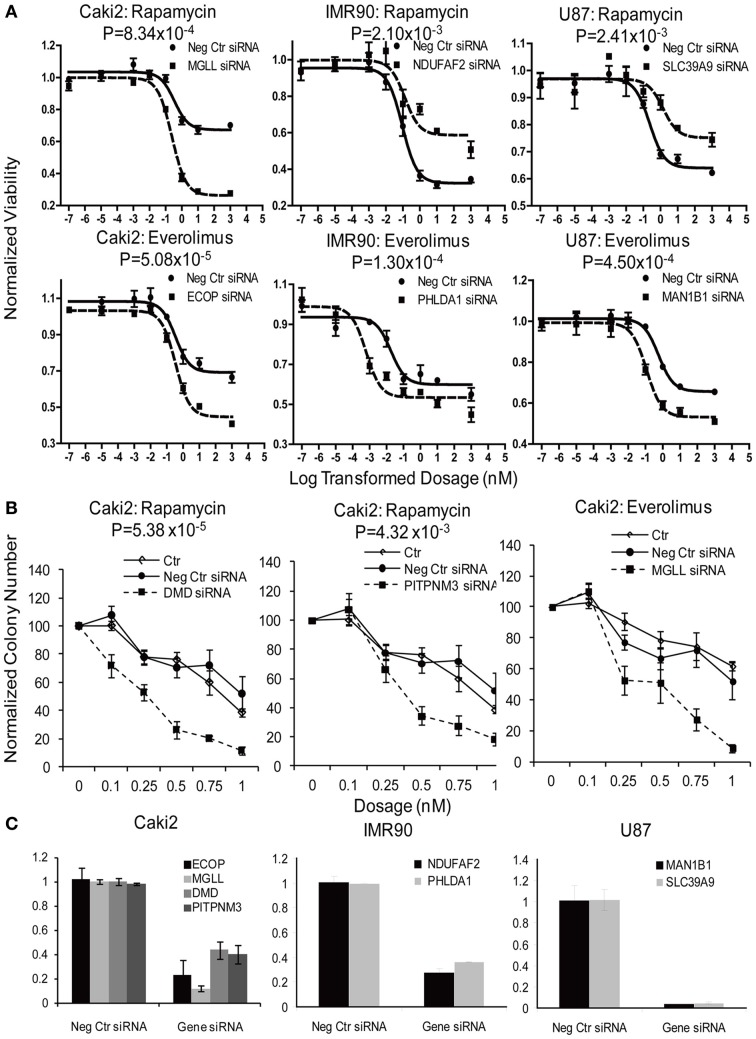
**Functional validation of candidate genes with siRNA knockdown in IMR90, U87 and Caki2 cell lines, followed by cytotoxicity assay (A) and colony formation assays (B)**. Data shown are representative experiments for selected genes in each cell line. siRNA knockdown for each individual gene (dashed line) were compared with negative control siRNA (solid line). **(C)**. Knockdown efficiency was determined by qRT-PCR. Experiments were repeated in triplicate with at least two independent experiments. Significant *P*-values are listed for every gene. Error bars indicate standard error of the mean (SEM) values. Significance of AUC values between the control and specific siRNA was determined by student *t*-test.

In summary, we selected 23 genes based on the strategy shown in Figure 3 to perform siRNA screening, followed by MTS and colony formation assays to determine the effect of these candidate genes on the cytotoxicity of mTOR inhibitors. We performed these studies using Caki2 renal carcinoma cells, U87 glioblastoma cells, and IMR90 primary fibroblast cells. The two cancer cell lines were chosen because mTOR inhibitors are used to treat glioblastoma and renal carcinoma. The IMR90 cell line was chosen to be a normal cell line used in the study. Eleven out of twenty-three genes were verified to have a significant impact on the cytotoxicity of Rapamycin and/or Everolimus using the MTS assays in at least one cell line. Figure 4A shows the data for representative genes with a significant influence on the cytotoxicity of each drug treatment in each cell line. Specifically, knockdown of 5 genes, *ECOP*, *MGLL*, *SLC39A9*, *ZNF765*, and *MAN1B1* sensitized the cells to Rapamycin and/or Everolimus in at least 2 cell lines (*P* < 0.05). Down-regulation of *NDUFAF2* and *SLC39A9* desensitized the cells to Rapamycin treatment in IMR90 and U87 cell lines, respectively (*P* < 0.05). Additional genes that significantly altered cell sensitivities are shown in Supplementary Figures S2, S3, S4 for each cell line. All of the genes that were functionally verified are listed in Table 2.

**Table 2:** **Summary of functional validation of candidate genes**.

	**Gene**	**Association**	**Cytotoxicity (siRNA KD)**			**Colony formation (siRNA KD)**	**miR-10a regulation**
			**Caki2**	**IMR90**	**U87**	**Caki2**	**Caki2**
mRNA Exp vs. AUC (13 genes)	BTG2	Exp vs. AUC (*R* = 0.27)		Rap↓			
	ECOP	Exp vs. AUC (*R* = 0.29)	Rap↓ Eve↓		Eve↓	Rap↓	
	FBXW7	Exp vs. AUC (*R* = 0.26)		Rap↓			Yes
	GIMAP1	Exp vs. AUC (*R* = −0.24)					Yes
	GIMAP6	Exp vs. AUC (*R* = −0.26)					Yes
	GIMAP7	Exp vs. AUC (*R* = −0.25)					Yes
	MGLL	Exp vs. AUC (*R* = 0.23)	Rap↓ Eve↓		Eve↓	Rap↓ Eve↓	Yes
	NDUFAF2	Exp vs. AUC (*R* = −0.24)		Rap↑			Yes
	PBX3	Exp vs. AUC (*R* = 0.28)					Yes
	PHLDA1	Exp vs. AUC (*R* = 0.24)		Eve↓			Yes
	SLC39A9	Exp vs. AUC and 3 way (*R* = 0.24)	Rap↓	Rap↓	Rap↑ Eve↑		
	STAU	Exp vs. AUC (*R* = 0.25)					Yes
	ZNF765	Exp vs. AUC (*R* = 0.25)		Rap↓	Rap↓ Eve↓		
SNP vs. AUC (4 genes)	ABCC1	SNP vs. AUC				Rap↓	
	MCPT2	SNP vs. AUC			Eve↓		
	BTNL2	SNP vs. AUC (non-synonymous)					
	PITPNM3	SNP vs. AUC (non-synonymous)				Rap↓	
Integrated analysis (Exp, SNP and AUC) (6 genes)	c9orf153	Integrated analysis (SNP)					
	GYPC	Integrated analysis (SNP)					
	JUN	Integrated analysis (SNP)					
	MAN1B1	Integrated analysis (SNP)	Rap↓ Eve↓		Rap↓ Eve↓		
	YARS2	Integrated analysis (*R* = −0.24)					
	DMD	Integrated analysis (*R* = 0.20)		Rap↓		Rap↓ Eve↓	

*“Rap” represents Rapamycin treatment; “Eve” represents Everolimus treatment. “↓” indicates siRNA knockdown of the gene sensitizes to the Rapamycin and/or Everolimus response (P < 0.05). “↑” indicates siRNA knockdown of gene desensitizes to the Rapamycin and/or Everolimus response (P < 0.05). “Yes” indicates miR-10a was verified to regulate gene expression. Blank indicates no significant change in cytotoxicity after knockdown*.

Furthermore, colony formation assays were also performed for the genes expressed in the Caki2 cell line, due to the relative ease of colony formation with this cell line compared to the other cell lines studied. We confirmed that knockdown of *ECOP* and *MGLL* significantly reduced colony formation as compared with the negative siRNA control (*P* < 0.05) after treatment with Rapamycin and/or Everolimus (Figures 4B,C), an observation that was consistent with the cytotoxicity assay results for the same cell line. In addition, even though the *ABCC1*, *PITPNM3*, and *DMD* genes were not verified by MTS assay, they were also shown to significantly decrease colony formation after Rapamycin or/and Everolimus treatment. However, we realize that the performance of colony formation assays using only the Caki2 cell line may be biased since not all the candidate genes were well expressed in this particular cell line. Overall, we functionally validated 13 out of 23 candidate genes selected from the GWAS analyses in at least one cell line with at least one assay (refer to Table 2).

#### Effect of miR-10a on cytotoxicity of rapamycin and everolimus and gene regulation

**Figure 5:**
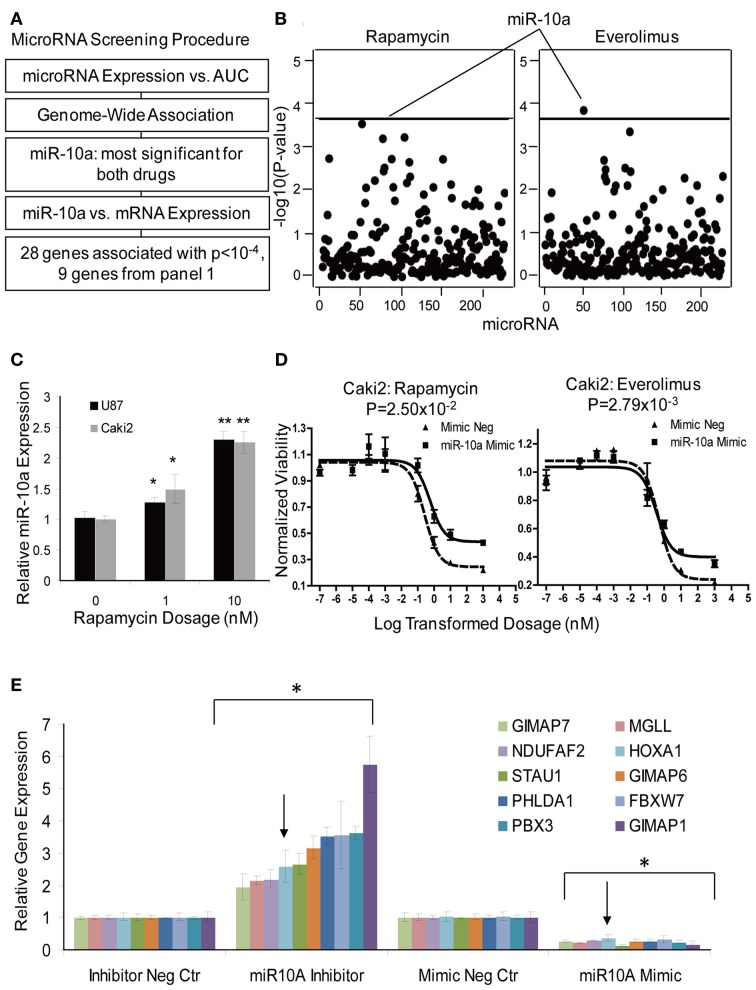
**MicroRNA screening and functional validation of miR-10a. (A)** Schematic diagram of the strategy used to select microRNAs for functional validation. **(B)** Genome-wide associations of microRNAs with AUC values for Rapamycin and Everolimus. MiR-10a was the most significant microRNA associated with AUC values for both Rapamycin and Everolimus. The x-axis represents 226 microRNA probes, and the y-axis represents the −log_10_(*P*-value) for the association of individual microRNA probe sets. A −log_10_(*P*-value) of 3.66 is highlighted with a horizontal line, indicating a *p*-value with genome-wide significance after Bonferroni correction for 228 tests. **(C)** Effect of Rapamycin on miR-10a Expression. MiR-10a expression was significantly enhanced by Rapamycin treatment compared with controls in Caki2 and U87 cell lines. **(D)** Effect of miR-10a on the cytotoxicity of Rapamycin and Everolimus. miR-10a overexpression (mimic) desensitized Caki2 cell to Rapamycin and Everolimus. **(E)** Gene regulation by miR-10a. miR-10a inhibitor “rescued” gene expression and mimic repressed gene expression in Caki2 cell line compared with inhibitor negative control or mimic negative control. The arrow indicates the positive control, the *HOXA1* gene. Experiments were performed in duplicate and were repeated 3 times. Error bars indicate mean ±SEM values. ^*^P < 0.05; ^**^P < 0.001.

MicroRNAs are a class of non-coding RNAs that regulate genes and/or protein expression by binding with mRNA to mediate mRNA degradation or block mRNA translation (Bartel, 2004, 2009). Therefore, microRNAs could also contribute to response to mTOR inhibitor effect. The microRNA screening procedure that we used is outlined graphically in Figure 5A. Briefly, 228 association tests were conducted between each microRNA expression probe and AUC values for both Everolimus and Rapamycin using the 262 cell lines for which we had both cytotoxicity and microRNA data sets. One microRNA expression probe, ILMN_3167552 (miR-10a), was highly associated with Everolimus AUC (*P* = 1.04 × 10^−4^, *R* = 0.2377), a value that reached genome-wide significance (Figure 5B). This same microRNA probe was also the most significant probe associated with Rapamycin AUC (*P* = 4.25 × 10^−4^, *R* = 0.2610). MiR-10a was further validated for its functional impact on cytotoxicity for both drugs in the Caki2 cell line. MiR-10a mimic significantly desensitized the cell to Rapamycin and Everolimus (*P* < 0.05), as shown in Figure 5D.

To further pursue the effect of microRNA on gene expression, we performed an association study of miR-10a with mRNAs whose expression levels were highly associated with Rapamycin and Everolimus cytotoxicity (*P* < 10^−4^). Thirty-one mRNA expression probe sets (28 genes) were determined to be highly associated with miR-10a expression (*P* < 10^−4^) (Supplementary Table S7). Specifically, 9 out of the 28 genes had already been included for siRNA screening, as highlighted in Supplementary Table S7. Therefore, those 9 genes were tested further for the effect of miR-10a on gene regulation. *HOXA1*, a known target for miR-10a, was used as a positive control gene (Mansfield et al., 2004; Garzon et al., 2006). All of the genes (including the positive control, *HOXA1*) were regulated by miR-10a in the same manner: miR-10a mimic significantly suppressed gene expression, while miR-10a inhibitor rescued gene expression (*P* < 0.05), as shown in Figure 5E. Four of those 9 genes (*PHLDA1*, *FBXW7*, *NDUFAF2*, and *MGLL*) were shown to have an effect on response to Rapamycin or Everolimus in one or more cell lines (Table 2). Down-regulation of 3 of the 4 genes (all except *NDUFAF2*) desensitized the cells to mTOR inhibitors. In addition, Rapamycin treatment significantly enhanced miR-10a expression in a dose dependent manner (Figure 5C, *P* < 0.05), suggesting that miR-10a might be suppressed by mTORC1 activation. We also tested the effect of miR-10a on mTORC1 signaling by measuring phosphorylated S6K and 4EBP1 and found that knockdown or overexpression of miR-10a did not result in a change of mTORC1 activity (data not shown).

## Discussion

mTOR inhibitors are a class of novel targeted agents that have shown promising results in cancer treatment. However, the response to mTOR inhibitors varies widely, ranging from lack of efficacy to the occurrence of undesired side effects. Genetic variation is one of the major factors that can play an important role in determining drug response (Wang et al., 2011). However, there are only few published data on the influence of genetics on mTOR inhibition effect. Examples include *CYP3A5* and *ABCB1* genotype effect on the pharmacokinetics of Rapamycin (Sirolimus) as an immunosuppressant for organ transplantation (Anglicheau et al., 2005; Mourad et al., 2005; Le Meur et al., 2006; Renders et al., 2007; Miao et al., 2008). Therefore, it would be important to identify and understand the biology underlying the possible role of genetic variation in determining drug response to mTOR inhibitors.

In this study, we took a genome-wide approach to screen for pharmacogenomic candidates that might alter the effect of mTOR inhibitors by taking advantage of extensive genomic data that we have obtained for 272 LCLs (SNPs, gene expression and microRNA expression), together with cytotoxicity data that we generated with two mTOR inhibitors, Rapamycin and Everolimus (Figures 1, 2). We used these two drugs to help inform the candidates identified between the drugs. This GWA analysis served as a hypothesis generating step, allowing us to screen for genomic candidates (SNP and genes) that showed strong associations with mTOR inhibitor-induced cytotoxicity. We then focused primarily on common candidates identified for both drugs. Genes such as *BTG2* and *FBXW7* that are known to affect the mTOR signaling pathway were also found to be associated with cytotoxicity of mTOR inhibitors in our study (Kim et al., 2008; Mao et al., 2008), suggesting that our association approach performed with 272 LCLs was capable of generating biologically relevant candidates for follow-up study.

The LCLs have limitations, as we have previously discussed (Niu et al., 2010). For example, EBV transformation can induce chromosomal instability and cellular changes (Sie et al., 2009). In addition, other factors such as cell growth rate and ATP level can have an effect on cytotoxicity (Choy et al., 2008). Since these cell lines do not necessarily represent the response of other types of tissues or cells (Dimas et al., 2009), we selected the top candidate genes based on our analyses to determine their contribution to variation in response to mTOR inhibitors. Two clinically relevant tumor cell lines, renal carcinoma (Caki2) and glioblastoma (U87), were selected for functional validation (Supplementary Figures S2, S3) since mTOR inhibitors are used as a treatment for these two types of tumors (Pantuck et al., 2006; Brugarolas et al., 2008; Cloughesy et al., 2008) and because our data suggested that these two cell lines were relatively more sensitive to mTOR inhibitor treatment. A fibroblast cell line (IMR90) was also included as a comparison to the tumor cell lines (Supplementary Figure S4). The two tumor cell lines, Caki2 and U87, tended to show similar results for several of the genes tested: *ECOP*, *MGLL*, and *MAN1B*. Our study showed that knockdown of these genes sensitized both Caki2 and U87 cells to mTOR inhibitors. *ECOP* (EGFR-coamplified and overexpressed protein), a gene which is amplified and overexpressed in at least a third of glioblastomas with EGFR amplification (Eley et al., 2002), is known to be a key regulator of NF-κB transcriptional activity that can contribute to cell survival (Park and James, 2005). IMR90 cells, on the other hand, seemed to be impacted by a different panel of genes, *BTG2, FBXW7, NDUFAF2, PHLDA1*, and *DMD*, whose knockdown did not have a significant impact in the two tumor cell lines, suggesting cell line-specific effects. Many of these genes have not been previously reported to interfere with the mTOR pathway except for *FBXW7* (F-box and WD repeat domain containing 7), which is known to target mTOR for degradation and which cooperates with PTEN for tumor suppression (Mao et al., 2008), and *BTG2* (B-cell translocation gene 2), which has been reported to inhibit AKT phosphorylation and mTOR signaling. Our results were compatible with the conclusion that down-regulation of FBXW7 restored the target for mTOR inhibitors, thus sensitizing cells to mTOR inhibitors, while knockdown of BTG2 activated the mTOR pathway which might cause the cells to become “addicted” to the mTOR pathway and, therefore, to benefit from mTOR inhibition. It is also worth noting that knockdown of *ZNF765* (zinc finger protein 765) was found to sensitize cells to mTOR inhibitors in both the IMR90 and U87 cell lines (Table 2). *ZNF765* is located on chromosome 19 and little is known with regard to its function. Therefore, its involvement in the mTOR pathway and response to mTOR inhibitors needs to be investigated further in the future.

Besides mRNA expression and SNPs, other genetic mechanisms, such as copy number variation, epigenetic effects (CpG methylation sites) and microRNAs might also influence response to mTOR inhibitor (Shenouda and Alahari, 2009). Despite the well-recognized importance of microRNAs and mTOR in cancer, very few studies have linked microRNAs with mTOR activity. MiR-99 was reported to mediate down-regulation of mTOR/FGFR3 and suppress tumor growth; miR-100 is known to inhibit mTOR signaling and enhance sensitivity to Everolimus in clear cell ovarian cancer (Nagaraja et al., 2010; Oneyama et al., 2011); and mTORC1 was recently reported to regulate miR-1 in skeletal myogenesis (Sun et al., 2010). Therefore, in this study we also attempted to determine whether microRNA might affect response to mTOR inhibitors. One microRNA (miR-10a) was shown to desensitize response to mTOR inhibitors (Figure 5D), and also affected the expression of several candidate genes that influenced sensitivity to mTOR inhibitors (Figure 5E). MiR-10a, a member of the miR-10 family members, maps to chromosome 17 upstream of the *HOX* gene cluster and putatively regulates expression of the *HOXA1*, *HOXA3*, and *HOXD10* genes (Garzon et al., 2006; Han et al., 2007). It is upregulated in glioblastoma, anaplastic astrocytomas and hepatocellular carcinoma (Ciafre et al., 2005; Gaur et al., 2007; Lund, 2010), and is known to be involved in the development of chronic and acute myeloid leukemia (Agirre et al., 2008; Jongen-Lavrencic et al., 2008). We also demonstrated that miR-10a can be induced by mTOR inhibitors and that genes highly associated with miR-10a were all negatively regulated by miR-10a. Based on this evidence, we hypothesize that mTOR inhibitors upregulate miR-10a expression, which in turn desensitizes cells to mTOR inhibitors response. This process probably occurs through the regulation of a set of genes whose expression levels are also critical in determining mTOR inhibitor response (Supplementary Table S7). Therefore, upregulation of miR-10a might be one mechanism for acquired resistance after Rapamycin therapy. Among the 9 genes that we tested, 5 had predicted binding sites for miR-10a. However, several genes were found experimentally to be negatively regulated by miR-10a, which was not consistent with the positive association values between miR-10a and mRNA expression (ex. FBXW7, *R* = 0.367; STAU1, *R* = 0.273; PHLDA1, *R* = 0.431, etc) observed in our LCLs. This might be due to the different cell specificity in terms of transcription regulation. We have also shown that inhibition of mTOR by Rapamycin upregulated miR-10a (Figure 5C), a process that might create a feedback loop resulting in desensitization of cells to mTOR inhibitors. However, the exact mechanisms by which miR-10a determines mTOR inhibitor response still need to be investigated in future studies.

## Conclusions

In summary, a pharmacogenomic approach based on the use of genomic data rich LCLs allowed us to identify a series of novel genetic candidates and a microRNAs that might contribute to variation in response to mTOR inhibitors. Functional validation of these candidates demonstrated the feasibility of utilizing this cell-line based model system and a GWA approach to generate hypotheses. These findings might help to enhance our understanding of the regulation of the mTOR pathway and of the mechanisms underlying variation in response to mTOR inhibitors. Obviously this study represents an early attempt to trying to identify biomarkers for response to mTOR inhibitors. These candidates can now be tested in clinical settings in future studies and, if confirmed, these studies could enhance our ability to individualize treatment with mTOR inhibitors.

## Authors′ contributions

Jing Jiang and Liewei Wang designed the study and wrote the manuscript. Jing Jiang and Pamela A. Long performed the experiments. Brooke L. Fridley, Ryan P. Abo, Abra Brisbin, and Anthony Batzler performed the statistical analyses. Ryan Abo conducted the bioinformatic analysis. Qiping Feng performed the miRNA array assay. All the authors read, revised the draft manuscript and approved the final version.

### Conflict of interest statement

The authors declare that the research was conducted in the absence of any commercial or financial relationships that could be construed as a potential conflict of interest.
