# Supplementary material for: Genome-wide association study for biomarker identification of Rapamycin and Everolimus using a lymphoblastoid cell line system
Source: Front Genet. 2013 Aug 30;4:166. doi: 10.3389/fgene.2013.00166 (PMC3757297; doi:10.3389/fgene.2013.00166)
Supplement: Supplementary file 1 [file DataSheet1.PDF]

Supplementary Figure Legends

Supplementary Figure S1. Effect of ethnicity on Rapamycin **(A)** and Everolimus **(B)** cytotoxicity (AUC values**)**. The x-axis indicates 3 ethnic groups: African-American (AA), Caucasian-American (CA) and Han Chinese-American (HCA). The y-axis indicates van der Waerden transformed Rapamycin AUC or log transformed Everolimus AUC values. Effect of gender on Rapamycin **(C)** and Everolimus **(D)** AUC values. The x-axis indicates genders: female (F) and male (M). The y-axis indicates van der Waerden transformed Rapamycin AUC or log transformed Everolimus AUC.

Supplementary Figure S2. Functional validation of candidate genes with siRNA knockdown in the Caki2 cell line, followed by cytotoxicity **(A)** and colony formation **(B)** assays. **(C)**. Data shown are for genes with significant impact on Caki2 phenotype(s) in addition to the representative genes shown in Fig 4. Knockdown efficiency was determined by qRT-PCR. Experiments performed in triplicate and were repeated at least twice. Error bars indicate SEM values. Significance was determined as $P<0.05$.

Supplementary Figure S3. (A). Functional validation of candidate genes with siRNA knockdown in the U87 cell line followed by cytotoxicity assays. Data shown are the genes with significant impact on U87 cytotoxicity in addition to the representative genes shown in Fig 4. **(B)**. Knockdown efficiency was determined by qRT-PCR. Experiments were performed in triplicate and were repeated at least twice. Error bars indicate SEM values. Significance was determined as $P<0.05$.

Supplementary Figure S4. (A). Functional validation of candidate genes with siRNA knockdown in the IMR90 cell line, followed by cytotoxicity assays Data shown are for genes with significant impact on IMR90 cytotoxicity in addition to the representative genes shown in Fig 4. **(B)**. Knockdown efficiency was determined by qRT-PCR. Experiments were performed in triplicate and were repeated at least twice. Error bars indicate SEM values. Significance was determined as $P<0.05$.

Supplementary Figure S1.

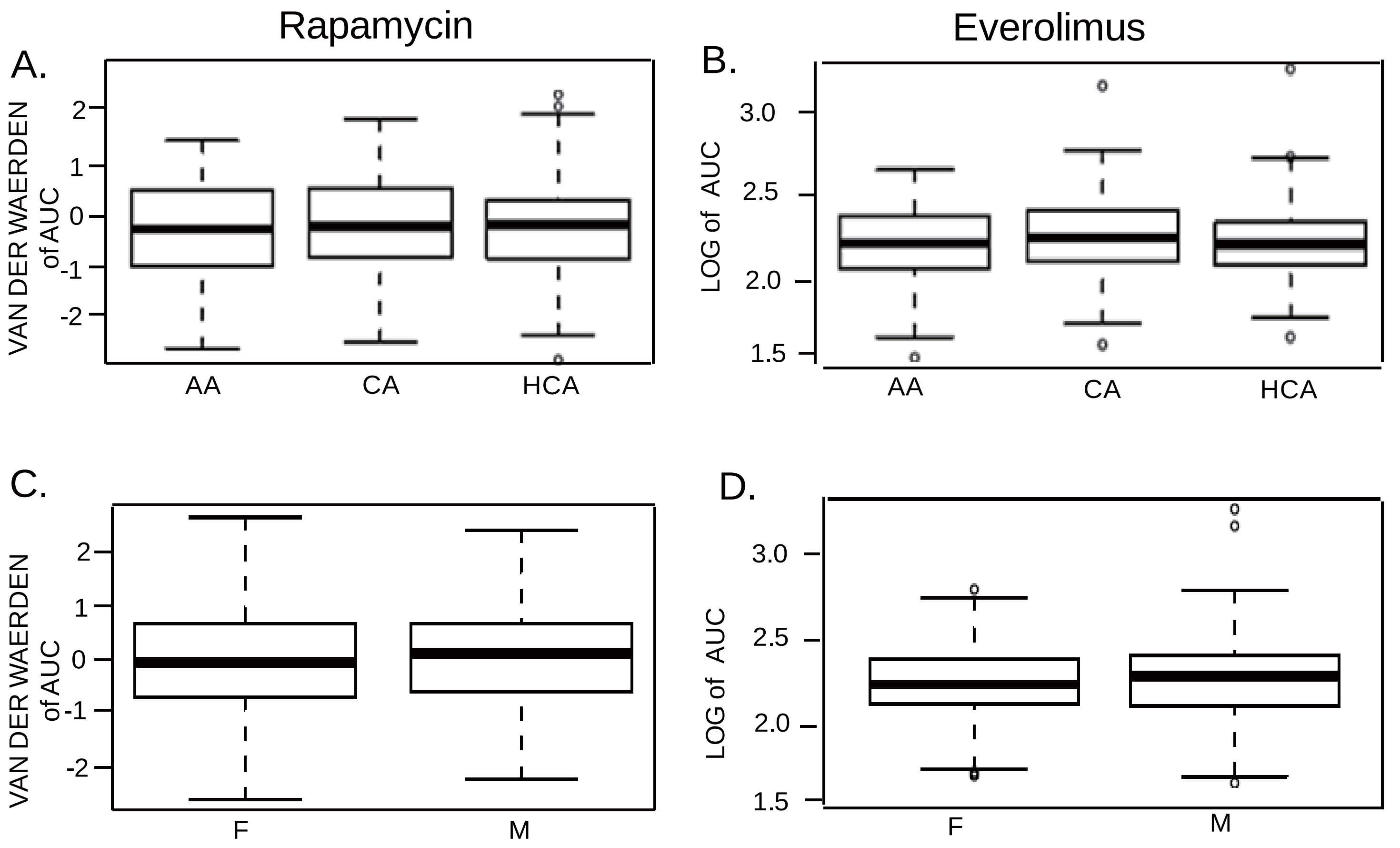

Supplementary Figure S2.

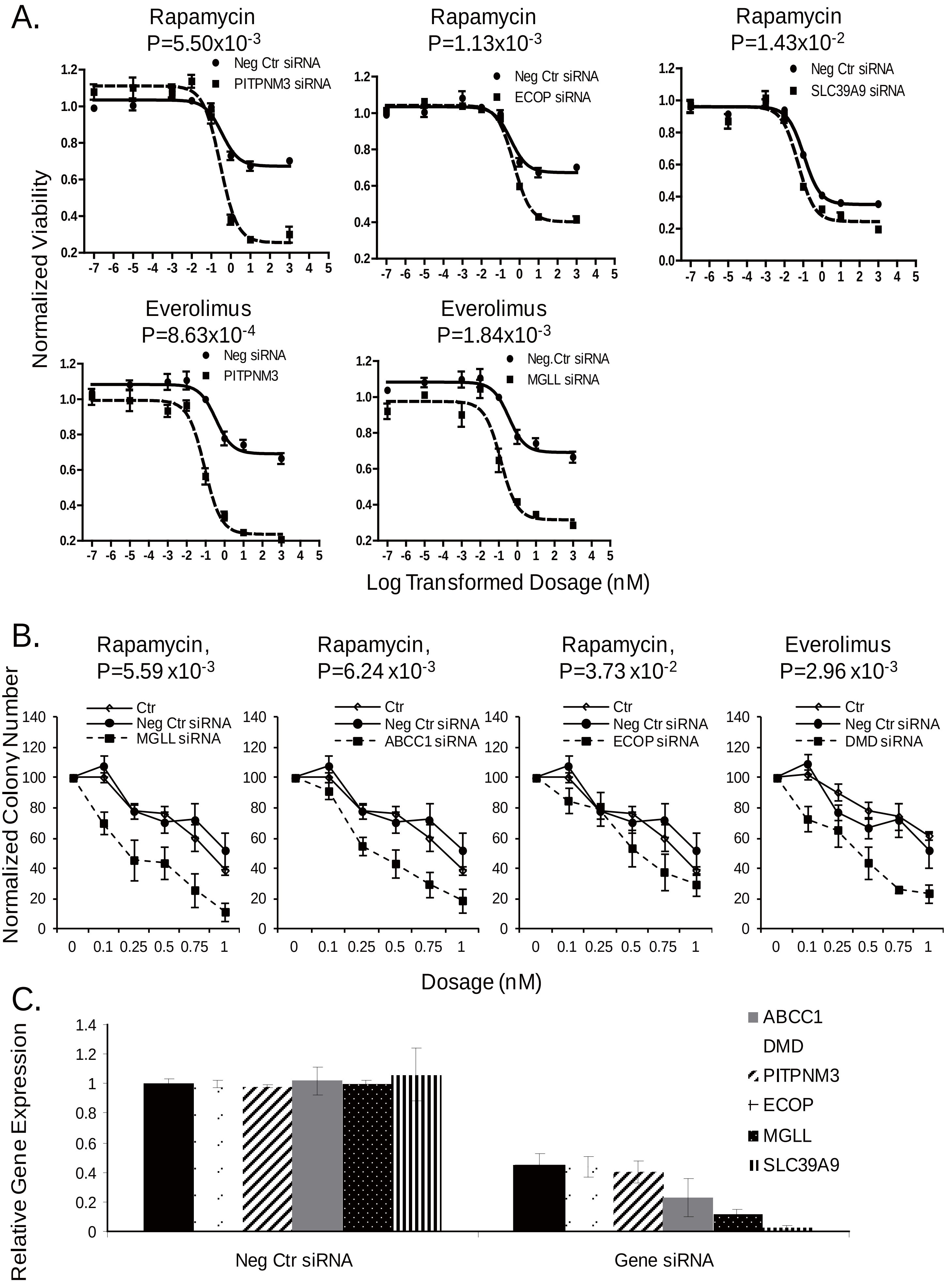

Supplementary Figure S3.

A.

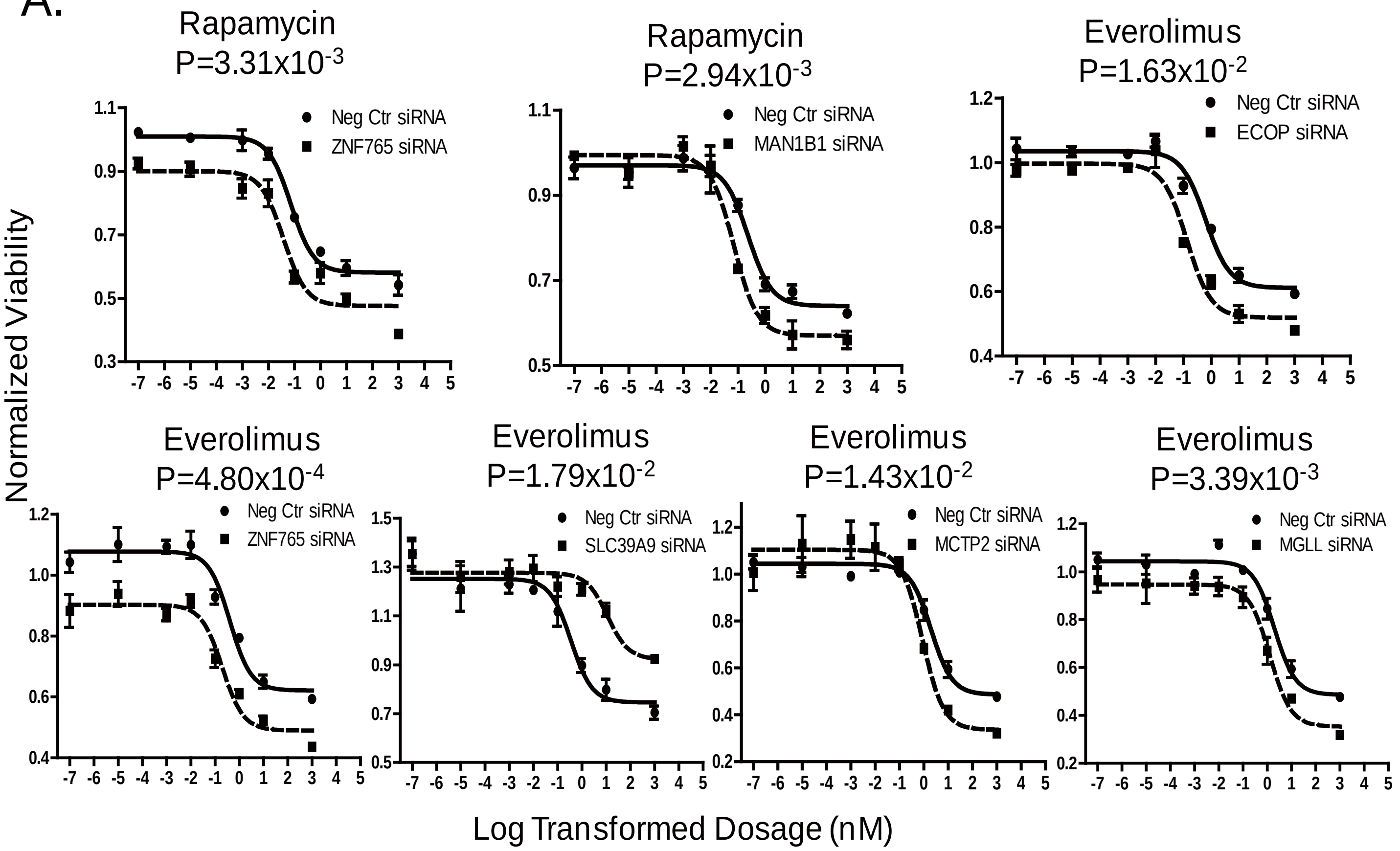

B.

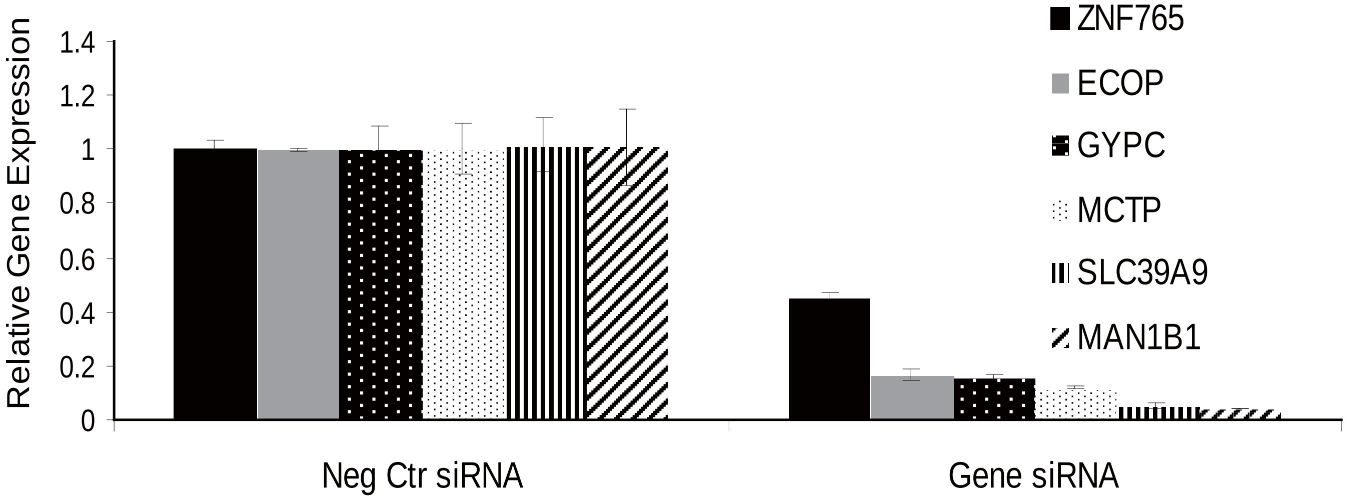

Supplementary Figure S4.

A.

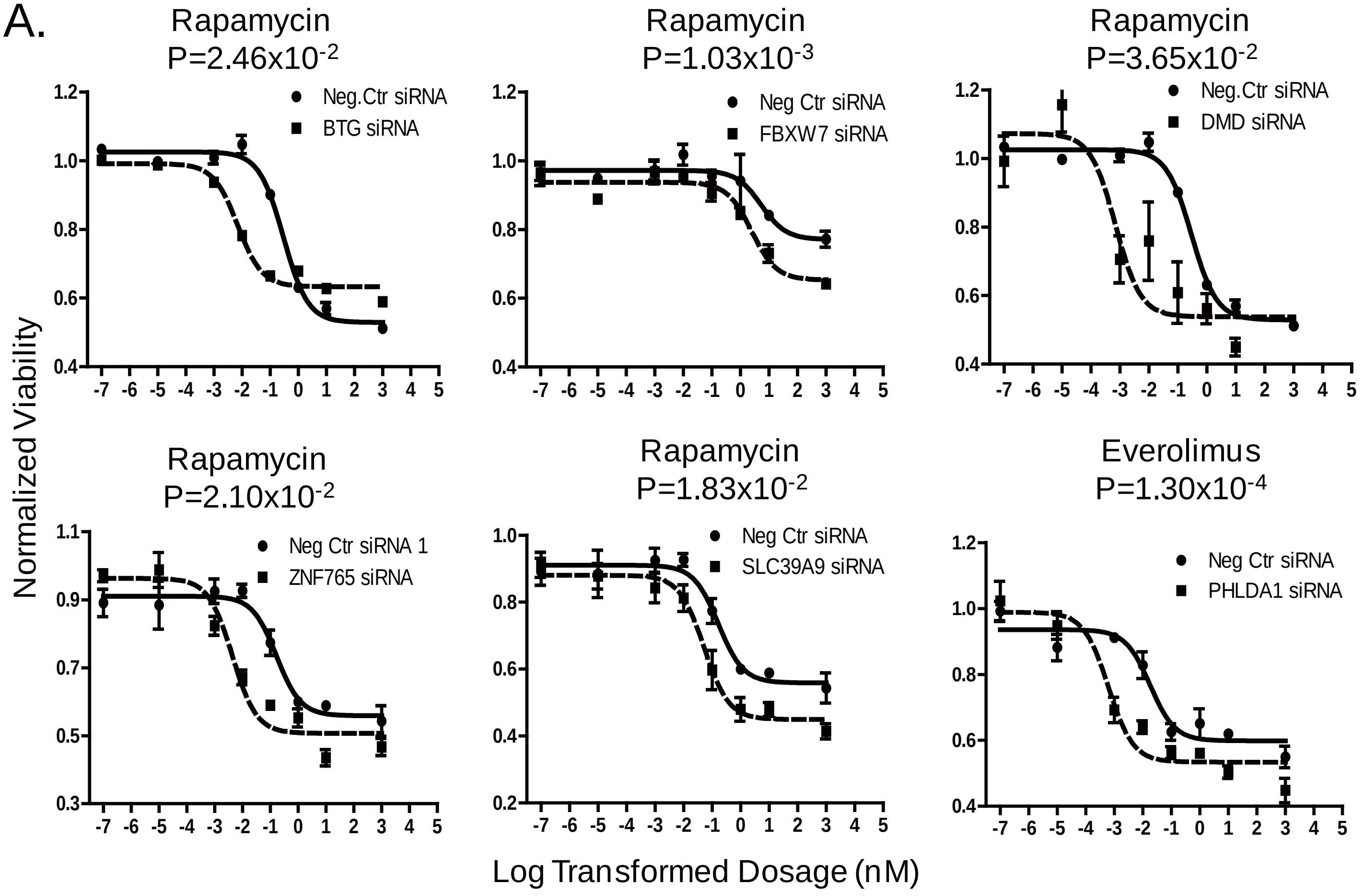

B.

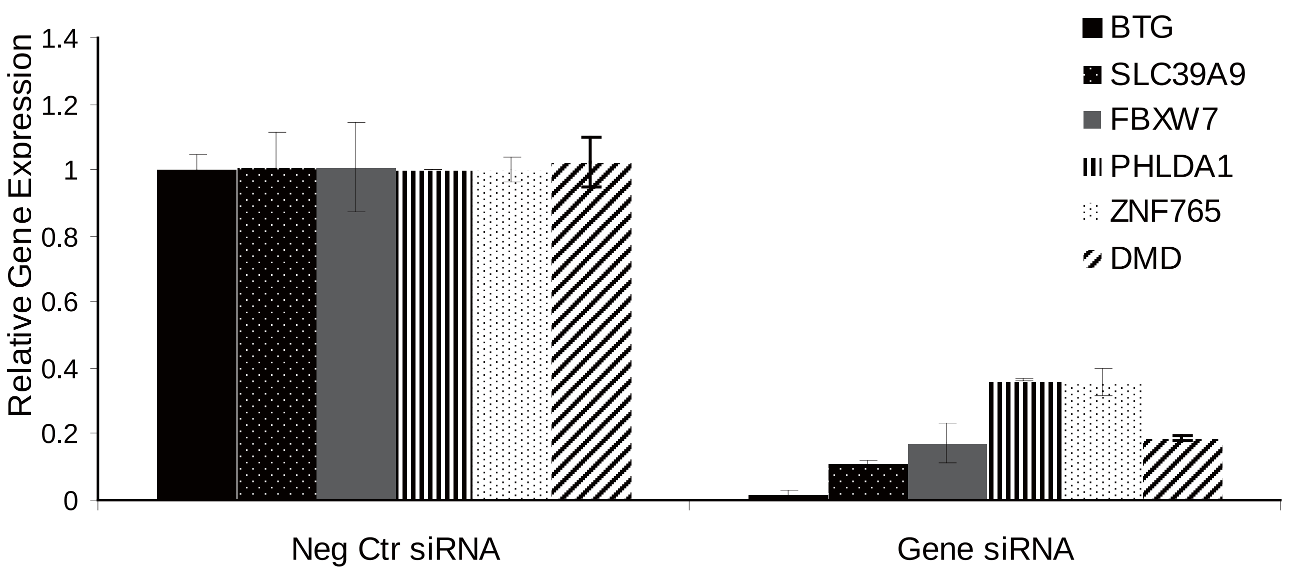

Supplementary Table S1. Genome-wide association of mRNA expression vs. Rapamycin AUC with $P<10^{-4}$.

RAP_EXP vs. Cyto (54k probe	p<0.00001					
**Probe.id**	**Gene Symbol**	**Chr**	**P**	**R**	**Bonferroni**	**Q**
229939_at	FLJ35220	17	1.12E-07	0.32	0.01	0.01
204082_at	PBX3	9	3.45E-06	0.28	0.19	0.08
201236_s_at	BTG2	1	6.97E-06	0.27	0.38	0.10
1562386_s_at	ZNF501	3	0.0000125	0.26	0.68	0.10
238513_at	PRRG4	11	0.0000155	0.26	0.85	0.10
202813_at	TARBP1	1	0.0000178	-0.26	0.97	0.10
230499_at	BIRC3	11	0.0000178	0.26	0.97	0.10
229419_at	FBXW7	4	0.0000195	0.26	1	0.10
202531_at	IRF1	5	0.0000218	0.25	1	0.10
214783_s_at	ANXA11	10	0.0000246	0.25	1	0.10
207320_x_at	STAU1	20	0.0000248	0.25	1	0.10
202932_at	YES1	18	0.0000255	0.25	1	0.10
212356_at	KIAA0323	14	0.0000284	0.25	1	0.10
1558943_x_at	ZNF765	19	0.0000349	0.25	1	0.10
203906_at	IQSEC1	3	0.000037	0.25	1	0.10
244564_at	FLJ37638	18	0.0000377	-0.25	1	0.10
228071_at	GIMAP7	7	0.0000391	-0.25	1	0.10
201952_at	ALCAM	3	0.0000391	0.25	1	0.10
217456_x_at	HLA-E	6	0.0000422	0.25	1	0.10
212312_at	BCL2L1	20	0.0000438	0.25	1	0.10
217996_at	PHLDA1	12	0.0000448	0.24	1	0.10
rs1325784	NCOA1	2	0.0000452	0.24	1	0.10
222656_at	UBE2W	8	0.000047	0.24	1	0.10
228355_s_at	NDUFAF2	5	0.0000475	-0.24	1	0.10
204730_at	RIMS3	1	0.0000519	0.24	1	0.10
203907_s_at	IQSEC1	3	0.0000582	0.24	1	0.10
229822_at	---	22	0.0000626	0.24	1	0.10
222445_at	SLC39A9	14	0.0000679	0.24	1	0.10
1558942_at	ZNF765	19	0.0000684	0.24	1	0.10
238567_at	SGPP2	2	0.0000686	0.24	1	0.10
244653_at	SETD7	4	0.0000714	-0.24	1	0.10
213770_at	KSR1	17	0.0000721	-0.24	1	0.10

204003_s_at	NUPL2	7	0.0000723	-0.24	1	0.10
214805_at	EIF4A1	17	0.0000738	-0.24	1	0.10
1552316_a_at	GIMAP1	7	0.0000816	-0.24	1	0.10
204794_at	DUSP2	2	0.0000817	0.24	1	0.10
210001_s_at	SOCS1	16	0.0000839	0.24	1	0.10
216701_at	C1orf68	1	0.000084	-0.24	1	0.10
200746_s_at	GNB1	1	0.0000861	0.24	1	0.10
226419_s_at	FLJ44342	17	0.0000864	-0.24	1	0.10
221796_at	NTRK2	9	0.0000871	0.24	1	0.10
238916_at	LOC400027	12	0.0000885	-0.24	1	0.10
210632_s_at	SGCA	17	0.0000903	0.24	1	0.10
208091_s_at	ECOP	1	0.0000942	0.23	1	0.10
203411_s_at	LMNA	1	0.0000995	0.23	1	0.10
226034_at	DUSP4	8	0.0000996	0.23	1	0.10
212050_at	WIPF2	17	0.0000998	0.23	1	0.10
204322_at	GOLIM4	3	0.000101	-0.23	1	0.10
225102_at	MGLL	3	0.000104	0.23	1	0.10

Chr.: Chromomosome; P represents the association p values; R represents the correlation coefficient; Q represents false discovery rate.

Supplementary Table S2. Genome-wide association of mRNA expression vs. Everolimus AUC for probe sets with P<10^{-4}.

EVE_EXP vs. Cyto (54k probe	p<0.00001					
**Probe ID**	**Gene Symbol**	**Chr**	**P**	**R**	**Bonferroni**	**Q**
229419_at	FBXW7	4	4E-07	0.30	0.02	0.01
214805_at	EIF4A1	17	1E-06	-0.29	0.05	0.01
208091_s_at	ECOP	1	1E-06	0.29	0.06	0.01
1554646_at	OSBPL1A	18	1E-06	0.29	0.06	0.01
238916_at	LOC400027	12	2E-06	-0.29	0.08	0.01
1558688_at	LOC441461	9	3E-06	0.28	0.14	0.02
217996_at	PHLDA1	12	4E-06	0.28	0.21	0.03
203759_at	ST3GAL4	11	5E-06	-0.27	0.26	0.03
216685_s_at	MTAP	9	5E-06	-0.27	0.29	0.03
1552316_a_at	GIMAP1	7	5E-06	-0.27	0.30	0.03
227333_at	ERI2	16	7E-06	0.27	0.39	0.03
229367_s_at	GIMAP6	7	1E-05	-0.26	0.53	0.04
202157_s_at	CUGBP2	10	1E-05	-0.26	0.67	0.05
1564362_x_at	ZNF843	16	1E-05	0.26	0.76	0.05
222445_at	SLC39A9	14	1E-05	0.26	0.81	0.05
201236_s_at	BTG2	1	2E-05	0.26	0.85	0.05
230499_at	BIR3C	11	2E-05	0.26	0.89	0.05
1557192_at	---	17	2E-05	0.26	1.00	0.05
205504_at	BTK	23	2E-05	-0.26	1.00	0.05
228980_at	RFFL	17	2E-05	0.26	1.00	0.05
1560225_at	CNR1	6	2E-05	0.25	1.00	0.05
228355_s_at	NDUFAF2	5	2E-05	-0.25	1.00	0.05
1565150_at	---	15	2E-05	0.25	1.00	0.05
226419_s_at	FLJ44342	17	2E-05	-0.25	1.00	0.05
244287_at	SFRS12	5	3E-05	-0.25	1.00	0.05
222040_at	HNRNPA1 /// LOC728844	12	3E-05	-0.25	1.00	0.05
207320_x_at	STAU1	20	3E-05	0.25	1.00	0.05
229939_at	FLJ35220	17	4E-05	0.25	1.00	0.06
228071_at	GIMAP7	7	4E-05	-0.25	1.00	0.06
225102_at	MGLL	3	4E-05	0.25	1.00	0.06
222457_s_at	LIMA1	12	4E-05	-0.24	1.00	0.06
209828_s_at	IL16	15	5E-05	-0.24	1.00	0.06
244564_at	FLJ37638	18	5E-05	-0.24	1.00	0.06
31637_s_at	NR1D1 /// THRA	17	5E-05	0.24	1.00	0.06
222729_at	FBXW7	4	5E-05	0.24	1.00	0.06
227287_at	CITED2	6	5E-05	-0.24	1.00	0.06

214368_at	RASGRP2	11	5E-05	-0.24	1.00	0.06
217079_at	---	13	5E-05	-0.24	1.00	0.07
238792_at	PCNX	14	6E-05	0.24	1.00	0.07
224813_at	WASL	7	6E-05	0.24	1.00	0.07
227402_s_at	UTP23	8	6E-05	-0.24	1.00	0.07
1555062_s_at	GTPBP3	19	6E-05	-0.24	1.00	0.07
218470_at	YARS2	12	6E-05	-0.24	1.00	0.07
1556914_at	BC031235	19	7E-05	0.24	1.00	0.07
208895_s_at	DDX18	2	7E-05	-0.24	1.00	0.07
222997_s_at	MRPS21	1	7E-05	-0.24	1.00	0.07
229147_at	---	4	7E-05	0.24	1.00	0.07
1563030_at	---	17	7E-05	0.24	1.00	0.07
222264_at	HNRNPUL2	11	8E-05	-0.24	1.00	0.07
217788_s_at	GALNT2	1	8E-05	0.24	1.00	0.08
206598_at	INS	11	8E-05	-0.24	1.00	0.08
224036_s_at	LMBR1	7	9E-05	0.24	1.00	0.08
208994_s_at	PPIG	2	9E-05	-0.24	1.00	0.08
225317_at	ACBD6	1	9E-05	0.23	1.00	0.08
1559993_at	SFXN3	10	1E-04	-0.23	1.00	0.08
1552315_at	GIMAP1	7	1E-04	-0.23	1.00	0.08

<u>Supplementary Table S3</u>. Genome-wide association of SNPs vs. Rapamycin AUC for SNPs with $P<10^{-4}$.

**Rap_SNP vs. Cytc**	**p<0.0001**								
**SNP ID**	**Chrom**	**Position**	**Gene**	**Location**	**MAF**	**P**	**R**	**Bonferroni**	**Q**
rs2063142	1	161319913	RGS5	NA	0.25	3.92E-06	-0.29	1	0.96
rs2841957	1	161319642	RGS4	flanking_5UTR	0.25	4.45E-06	-0.28	1	0.96
rs17664713	15	93119590	MCTP2	flanking_3UTR	0.15	4.70E-06	-0.28	1	0.96
rs1873283	15	27125794	APBA2	intron	0.29	6.46E-06	-0.28	1	0.96
rs12636856	3	20111963	PCAF	NA	0.24	7.13E-06	-0.28	1	0.96
rs1334346	6	73249872	RIMS1	NA	0.36	8.24E-06	-0.28	1	0.96
rs2781564	6	73234584	RIMS1	NA	0.37	8.42E-06	-0.28	1	0.96
rs6907281	6	72044931	OGFRL1	NA	0.37	9.79E-06	-0.27	1	0.96
rs7325009	13	97679564	FARP1	NA	0.43	1.06E-05	-0.27	1	0.96
rs4394887	12	2520241	CACNA1C	intron	0.16	1.10E-05	0.27	1	0.96
rs6914937	6	72036050	OGFRL1	NA	0.37	1.15E-05	-0.27	1	0.96
rs9346458	6	72067684	OGFRL1	NA	0.38	1.25E-05	-0.27	1	0.96
rs694243	11	64928773	FKSG44	intron	0.40	1.27E-05	0.27	1	0.96
rs1507739	1	161353709	RGS5	NA	0.24	1.31E-05	-0.27	1	0.96
rs2841984	1	161352820	RGS5	flanking_3UTR	0.26	1.32E-05	-0.27	1	0.96
rs2643358	15	99789558	PCSK6	NA	0.15	1.52E-05	0.27	1	0.96
rs2219206	2	126510268	GYPC	NA	0.05	1.56E-05	0.27	1	0.96
rs10848675	12	2530109	CACNA1C	intron	0.09	1.60E-05	0.27	1	0.96
rs2239105	12	2493926	CACNA1C	NA	0.31	1.68E-05	0.27	1	0.96
rs2291928	12	120500	IQSEC3	intron	0.29	1.70E-05	0.27	1	0.96
rs2670625	4	90197890	FAM13A1	flanking_5UTR	0.25	1.83E-05	0.27	1	0.96
rs1885677	6	72080854	OGFRL1	NA	0.35	1.92E-05	-0.26	1	0.96
rs1885676	6	72089847	OGFRL1	NA	0.35	1.99E-05	-0.27	1	0.96
rs2239104	12	2493804	CACNA1C	intron	0.28	2.01E-05	0.26	1	0.96
rs2696851	16	84881876	BC043214	NA	0.06	2.04E-05	-0.26	1	0.96
rs4148330	16	15949269	ABCC1	flanking_5UTR	0.49	2.27E-05	-0.26	1	0.96
rs11048543	12	26517905	ITPR2	intron	0.14	2.32E-05	-0.26	1	0.96
rs4263048	19	58469142	VN1R4	intron	0.20	2.35E-05	0.26	1	0.96
rs9558712	13	105646375		intron	0.47	2.43E-05	-0.26	1	0.96
rs6665029	1	34813578	GJB5	flanking_5UTR	0.11	2.51E-05	-0.26	1	0.96
rs2304474	16	9443019	GRIN2A	flanking_3UTR	0.16	2.73E-05	-0.26	1	0.96
rs2076523	6	32478813	BTNL2	coding	0.39	2.77E-05	-0.26	1	0.96
rs4867154	5	27758844	CDH9	flanking_5UTR	0.37	2.86E-05	0.26	1	0.96

rs2073894	19	41193285	ALKBH6	intron	0.46	2.98E-05	-0.26	1	0.96
rs2073896	19	41200232	CLIPR-59	intron	0.49	3.31E-05	-0.26	1	0.96
rs10492650	13	105641808	EFNB2	flanking_3UTR	0.43	3.31E-05	-0.26	1	0.96
rs17099886	12	62567319	SRGAP1	NA	0.10	3.40E-05	-0.26	1	0.96
rs2269520	22	48147268	FLJ44385	flanking_5UTR	0.33	3.52E-05	0.26	1	0.96
rs11629532	15	96084557	ARRDC4	NA	0.21	3.66E-05	0.26	1	0.96
rs12227580	12	2511938	CACNA1C	NA	0.07	3.77E-05	0.26	1	0.96
rs12872413	13	105641766	EFNB2	flanking_3UTR	0.43	3.84E-05	-0.26	1	0.96
rs11075286	16	15935225	ABCC1	flanking_5UTR	0.45	3.93E-05	-0.26	1	0.96
rs2239109	12	2499072	CACNA1C	intron	0.27	3.97E-05	0.25	1	0.96
rs10846216	12	15631561	PTPRO	flanking_3UTR	0.20	4.15E-05	0.25	1	0.96
rs12905214	15	67412324	PAQR5	NA	0.09	4.17E-05	0.25	1	0.96
rs10918620	1	165314744	GPA33	NA	0.16	4.18E-05	0.25	1	0.96
rs3806156	6	32481676	BTNL2	intron	0.38	4.31E-05	-0.25	1	0.96
rs2280201	10	71550196	H2AFY2	UTR	0.21	4.43E-05	0.25	1	0.96
rs7595408	2	231029204	SP100	intron	0.49	4.49E-05	-0.25	1	0.96
rs1978613	19	58460149	VN1R4	flanking_5UTR	0.37	4.52E-05	-0.26	1	0.96
rs4142303	20	87841	DEFB128	NA	0.42	4.65E-05	0.25	1	0.96
rs7694207	4	98099432	MGC46496	flanking_3UTR	0.26	4.73E-05	0.25	1	0.96
rs7135745	12	53153518	FAM112B	NA	0.11	4.80E-05	-0.25	1	0.96
rs10780752	9	88016129	C9orf153	NA	0.31	4.90E-05	-0.25	1	0.96
rs10918618	1	165312136	GPA33	intron	0.16	4.91E-05	0.25	1	0.96
rs1795731	4	90195905	FAM13A1	intron	0.24	4.98E-05	0.25	1	0.96
rs16887552	4	12383092		flanking_5UTR	0.24	5.00E-05	-0.25	1	0.96
rs11062260	12	2517044	CACNA1C	intron	0.06	5.11E-05	0.26	1	0.96
rs10212561	3	71208294	FOXP1	NA	0.14	5.35E-05	0.25	1	0.96
rs12812	11	1934128	MRPL23	coding	0.10	5.35E-05	-0.26	1	0.96
rs10515734	5	155626835	SGCD	intron	0.12	5.67E-05	-0.25	1	0.96
rs1325784	13	105649007	EFNB2	flanking_3UTR	0.46	5.70E-05	-0.25	1	0.96
rs9555232	13	105646287	FLJ35220	intron	0.46	5.70E-05	-0.25	1	0.96
rs9301121	13	105648511		intron	0.46	5.70E-05	-0.25	1	0.96
rs7324946	13	105649071		NA	0.46	5.70E-05	-0.25	1	0.96
rs2693026	2	15844044	DDX1	NA	0.29	5.72E-05	0.25	1	0.96
rs1536235	13	105648472		NA	0.47	5.84E-05	-0.25	1	0.96
rs7202570	16	9462870	AF205217	flanking_5UTR	0.17	5.90E-05	-0.25	1	0.96
rs756175	4	90218064	FAM13A1	flanking_5UTR	0.25	6.04E-05	0.25	1	0.96
rs4144048	2	126509995	GYPC	flanking_5UTR	0.05	6.20E-05	0.25	1	0.96

rs17035581	4	106574145	PPA2	flanking_3UTR	0.09	6.20E-05	-0.25	1	0.96
rs9557916	13	102150614	LOC196541	NA	0.33	6.24E-05	0.25	1	0.96
rs9858020	3	197704191	RNF168	intron	0.47	6.32E-05	0.25	1	0.96
rs218869	8	23692226		flanking_3UTR	0.37	6.52E-05	0.25	1	0.96
rs1209049	6	73326765	KCNQ5	flanking_5UTR	0.28	6.60E-05	-0.25	1	0.96
rs1469440	19	58451333	ZNF677	NA	0.36	6.60E-05	-0.25	1	0.96
rs12133648	1	165311151	GPA33	intron	0.29	6.62E-05	0.25	1	0.96
rs9315538	13	37508936	TRPC4	intron	0.07	6.71E-05	-0.25	1	0.96
rs10241701	7	9204051	NXPH1	NA	0.34	6.72E-05	0.25	1	0.96
rs512715	11	64947784	TncRNA	NA	0.39	6.88E-05	0.25	1	0.96
rs4806248	19	41206931	CLIPR-59	intron	0.48	6.92E-05	-0.25	1	0.96
rs6835031	4	90214448	FAM13A1	NA	0.25	7.02E-05	0.25	1	0.96
rs10256687	7	79681423	GNAI1	NA	0.39	7.19E-05	0.25	1	0.96
rs10918616	1	165311957	GPA33	intron	0.29	7.23E-05	0.25	1	0.96
rs10918619	1	165314691	GPA33	NA	0.29	7.23E-05	0.25	1	0.96
rs9916344	17	6338937	PITPNM3	NA	0.45	7.27E-05	0.25	1	0.96
rs12655476	5	155632160	SGCD	NA	0.12	7.29E-05	-0.25	1	0.96
rs7832468	8	8484184	CLDN23	NA	0.47	7.36E-05	-0.25	1	0.96
rs12231350	12	2505451	CACNA1C	intron	0.22	7.61E-05	0.25	1	0.96
rs3809835	17	6347607	PITPNM3	NA	0.30	7.73E-05	-0.25	1	0.96
rs12565684	1	27533624	C1orf160	intron	0.27	7.81E-05	0.25	1	0.96
rs10501576	11	84254292	DLG2	intron	0.16	7.91E-05	0.25	1	0.96
rs328239	14	80277041	C14orf145	intron	0.48	7.97E-05	0.25	1	0.96
rs10870177	9	139107171	MAN1B1	intron	0.16	8.04E-05	-0.24	1	0.96
rs10918621	1	165317716	GPA33	intron	0.20	8.09E-05	0.24	1	0.96
rs4497499	12	62499370	TMEM5	NA	0.30	8.14E-05	-0.25	1	0.96
rs7318834	13	108710500	MYR8	flanking_3UTR	0.05	8.19E-05	-0.24	1	0.96
rs7522826	1	57899105	DAB1	NA	0.15	8.33E-05	0.24	1	0.96
rs7449744	6	51842122	PKHD1	flanking_3UTR	0.45	8.37E-05	-0.24	1	0.96
rs17038464	1	12709031	LOC126767	3UTR	0.37	8.56E-05	-0.24	1	0.96
rs1389932	5	116549238		intron	0.24	8.58E-05	0.24	1	0.96
rs2904976	11	64941963	TncRNA	flanking_3UTR	0.27	8.61E-05	0.24	1	0.96
rs17732246	15	93120790	MCTP2	NA	0.10	8.63E-05	-0.25	1	0.96
rs588849	23	102893254	TMEM31	NA	0.18	8.76E-05	0.24	1	0.96
rs4535089	2	41922802	LOC91461	flanking_5UTR	0.42	8.91E-05	0.24	1	0.96
rs12742115	1	26952518	ARID1A	intron	0.05	8.94E-05	0.24	1	0.96
rs11048538	12	26509526	ITPR2	NA	0.13	9.05E-05	-0.24	1	0.96

rs7116710	11	15218897	INSC	NA	0.40	9.06E-05	-0.24	1	0.96
rs10797668	1	179302377	MR1	flanking_3UTR	0.33	9.09E-05	0.24	1	0.96
rs3890739	7	79682046	GNAI1	NA	0.28	9.10E-05	0.24	1	0.96
rs3949912	1	59195691	JUN	flanking_5UTR	0.43	9.17E-05	-0.24	1	0.96
rs11661096	18	42379165	LOXHD1	NA	0.07	9.23E-05	-0.24	1	0.96
rs1426723	18	28394022	FAM59A	flanking_5UTR	0.49	9.26E-05	0.24	1	0.96
rs7182320	15	81184806	AP3B2	flanking_5UTR	0.24	9.28E-05	0.24	1	0.96
rs2702449	4	179638427	AGA	flanking_5UTR	0.37	9.34E-05	0.24	1	0.96
rs4846052	1	11780538	MTHFR	intron	0.46	9.38E-05	-0.25	1	0.96
rs819194	8	23797657	STC1	NA	0.47	9.43E-05	0.25	1	0.96
rs17293755	10	1343714	ADARB2	intron	0.09	9.74E-05	-0.24	1	0.96
rs7543260	1	59208276	JUN	flanking_5UTR	0.18	9.78E-05	-0.24	1	0.96
rs11170917	12	53152255	FAM112B	intron	0.15	9.82E-05	-0.24	1	0.96
rs1842033	3	27943951	MGC61571	NA	0.07	9.84E-05	0.24	1	0.96
rs2544527	2	15843619	DDX1	NA	0.20	9.89E-05	0.25	1	0.96
rs2294777	6	148837165	SASH1	coding	0.05	9.91E-05	0.24	1	0.96
SNP_A-1791403	6	148847826	SASH1	intron	0.05	9.91E-05	0.24	1	0.96
rs1969888	13	105648715	EFNB2	flanking_3UTR	0.46	9.92E-05	-0.24	1	0.96
rs327465	14	80299793	C14orf145	NA	0.47	9.99E-05	0.24	1	0.96
rs570046	18	53295430	ONECUT2	NA	0.44	9.99E-05	-0.24	1	0.96

Supplementary Table S4. **Genome-wide association of SNPs vs. Everolimus AUC for SNPs with $P<10^{-4}$.**

SNP ID	Chromosome	Position	Gene	Location	MAF	P	R	Bonferroni	Q
rs218869	8	23692226	NA	NA	0.37	3.61E-06	0.29	1	0.97
rs7694207	4	98099432	MGC46496	flanking_3UTR	0.26	3.98E-06	0.28	1	0.97
rs10987149	9	127897672	PBX3	flanking_3UTR	0.32	4.57E-06	0.28	1	0.97
rs12932018	16	83354104	USP10	NA	0.25	5.78E-06	0.28	1	0.97
rs2702449	4	179638427	AGA	flanking_5UTR	0.37	7.35E-06	0.28	1	0.97
rs16887552	4	12383092	NA	NA	0.24	8.25E-06	-0.28	1	0.97
rs2832270	21	29516403	C21orf7	flanking_3UTR	0.14	8.39E-06	0.28	1	0.97
rs1460196	18	48469410	DCC	intron	0.08	9.25E-06	0.27	1	0.97
rs4148330	16	15949269	ABCC1	flanking_5UTR	0.49	9.80E-06	-0.27	1	0.97
rs13272072	8	23689236	STC1	flanking_3UTR	0.10	9.87E-06	0.27	1	0.97
rs17664713	15	93119590	MCTP2	flanking_3UTR	0.15	1.55E-05	-0.27	1	0.97
rs11048543	12	26517905	ITPR2	intron	0.14	1.73E-05	-0.27	1	0.97
rs7786803	7	101012950	EMID2	flanking_3UTR	0.17	1.98E-05	0.26	1	0.97
rs10870177	9	139107171	MAN1B1	intron	0.16	1.99E-05	-0.26	1	0.97
rs11981141	7	137735053	NA	NA	0.08	1.99E-05	0.26	1	0.97
rs9353750	6	91416161	MAP3K7	flanking_5UTR	0.41	2.02E-05	-0.26	1	0.97
rs11734813	4	77759770	SHROOM3	NA	0.07	2.04E-05	0.26	1	0.97
rs2271165	17	4596127	ZMYND15	3UTR	0.15	2.16E-05	0.26	1	0.97
rs7543260	1	59208276	JUN	flanking_5UTR	0.18	2.35E-05	-0.26	1	0.97
rs2832267	21	29513533	BACH1	NA	0.11	2.39E-05	0.26	1	0.97
rs1602517	3	193287854	FGF12	flanking_3UTR	0.44	2.39E-05	-0.26	1	0.97
rs1862486	19	59631853	TTYH1	intron	0.10	2.39E-05	0.26	1	0.97
rs5011574	12	67053394	MDM1	flanking_5UTR	0.26	2.43E-05	0.26	1	0.97
rs1252068	1	175868691	LZTR2	flanking_3UTR	0.17	3.02E-05	-0.26	1	0.97
rs8086577	18	48463367	DCC	NA	0.08	3.06E-05	0.26	1	0.97
rs2296630	10	129092719	DOCK1	intron	0.20	3.13E-05	0.26	1	0.97
rs6951882	7	8892313	NXPH1	flanking_3UTR	0.13	3.30E-05	-0.26	1	0.97
rs1480577	2	161306456	RBMS1	flanking_5UTR	0.25	3.36E-05	-0.26	1	0.97
rs10780752	9	88016129	C9orf153	NA	0.31	3.49E-05	-0.26	1	0.97
rs3823419	6	31208980	PSORS1C1	flanking_3UTR	0.25	3.55E-05	-0.26	1	0.97
rs6720147	2	161394360	RBMS1	NA	0.48	3.57E-05	0.26	1	0.97

rs6029138	20	38546320	MAFB	flanking_3UTR	0.19	3.59E-05	0.26	1	0.97
rs6830552	4	98109064	C4orf37	NA	0.36	3.60E-05	0.26	1	0.97
rs6834049	4	98125318	MGC46496	flanking_3UTR	0.36	3.60E-05	0.26	1	0.97
rs11180333	12	73688157	KCNC2	NA	0.46	3.66E-05	-0.26	1	0.97
rs438660	7	137715870	NA	NA	0.09	3.70E-05	0.26	1	0.97
rs451408	7	137716210	NA	NA	0.09	3.70E-05	0.26	1	0.97
rs427508	7	137717716	TRIM24	flanking_5UTR	0.09	3.70E-05	0.26	1	0.97
rs7967521	12	123882723	SCARB1	intron	0.40	3.85E-05	0.26	1	0.97
rs1602518	3	193287456	NA	NA	0.44	3.97E-05	-0.25	1	0.97
rs4075537	10	133090374	TCERG1L	flanking_5UTR	0.29	3.98E-05	0.25	1	0.97
rs1504274	7	68372905	AUTS2	flanking_5UTR	0.23	4.06E-05	-0.25	1	0.97
rs7529907	1	175905638	LZTR2	flanking_3UTR	0.16	4.08E-05	-0.25	1	0.97
rs3823418	6	31208921	PSORS1C1	flanking_3UTR	0.24	4.16E-05	-0.25	1	0.97
rs12902244	15	99789578	PCSK6	intron	0.09	4.24E-05	-0.25	1	0.97
rs2892333	10	1386155	ADARB2	intron	0.27	4.32E-05	-0.25	1	0.97
rs3823417	6	31208848	PSORS1C1	flanking_3UTR	0.28	4.41E-05	-0.25	1	0.97
rs1252077	1	175882217	SEC16B	NA	0.17	4.53E-05	-0.26	1	0.97
rs6835198	4	98122021	C4orf37	NA	0.36	4.55E-05	0.25	1	0.97
rs13102764	4	47125684	GABRB1	flanking_3UTR	0.48	4.59E-05	-0.25	1	0.97
rs17732246	15	93120790	MCTP2	NA	0.10	4.63E-05	-0.26	1	0.97
rs7433483	3	197701435	RNF168	NA	0.40	4.87E-05	0.25	1	0.97
rs8037827	15	33521840	ATPBD4	intron	0.25	5.04E-05	0.25	1	0.97
rs4242548	8	1867937	ARHGEF10	intron	0.46	5.59E-05	-0.25	1	0.97
rs6595143	5	117897073	DTWD2	flanking_3UTR	0.36	5.63E-05	0.25	1	0.97
rs12676599	8	142814556	FLJ43860	flanking_5UTR	0.49	5.66E-05	0.25	1	0.97
rs9858020	3	197704191	RNF168	intron	0.47	6.05E-05	0.25	1	0.97
rs11648730	16	9715078	GRIN2A	NA	0.16	6.06E-05	-0.25	1	0.97
rs4649309	1	231588888	NA	NA	0.27	6.07E-05	0.25	1	0.97
rs1252076	1	175884273	FAM5B	NA	0.16	6.13E-05	-0.25	1	0.97
rs11075286	16	15935225	ABCC1	flanking_5UTR	0.45	6.32E-05	-0.25	1	0.97
rs7167641	15	33537309	ATPBD4	intron	0.14	6.43E-05	0.25	1	0.97
rs17191541	14	96711131	VRK1	flanking_3UTR	0.13	6.46E-05	0.25	1	0.97
rs1543434	20	55442174	RNPC1	flanking_3UTR	0.06	6.52E-05	-0.25	1	0.97
rs430435	7	137724670	TRIM24	flanking_5UTR	0.16	6.69E-05	0.25	1	0.97
rs17138777	10	16614364	C1QL3	NA	0.10	6.73E-05	0.25	1	0.97

rs1447294	8	128506868	NA	NA	0.32	6.94E-05	-0.25	1	0.97
rs1470899	11	83663719	DLG2	intron	0.47	7.08E-05	-0.25	1	0.97
rs357361	7	137704985	TRIM24	flanking_5UTR	0.09	7.14E-05	0.25	1	0.97
rs7771544	6	162007040	PARK2	NA	0.05	7.17E-05	-0.25	1	0.97
rs11629532	15	96084557	ARRDC4	NA	0.21	7.24E-05	0.25	1	0.97
rs206937	6	34440157	NUDT3	intron	0.18	7.24E-05	-0.25	1	0.97
rs10016319	4	82719121	RASGEF1B	NA	0.35	7.34E-05	0.25	1	0.97
rs31075	16	53914739	IRX6	flanking_5UTR	0.36	7.37E-05	-0.25	1	0.97
rs17053273	8	25179409	DOCK5	NA	0.18	7.41E-05	0.25	1	0.97
rs10999854	10	72936342	CDH23	intron	0.47	7.54E-05	-0.25	1	0.97
rs4316216	9	88023285	C9orf153	flanking_3UTR	0.28	7.66E-05	-0.25	1	0.97
rs16845258	2	161431125	RBMS1	NA	0.26	7.67E-05	0.25	1	0.97
rs834063	7	136878084	DGKI	intron	0.10	7.77E-05	-0.25	1	0.97
rs150745	16	62006171	CDH11	NA	0.31	8.32E-05	0.24	1	0.97
rs3898039	15	33514657	ATPBD4	intron	0.25	8.43E-05	0.24	1	0.97
rs10155173	4	47116408	GABRB1	intron	0.49	8.48E-05	-0.24	1	0.97
rs1606035	8	25168872	DOCK5	NA	0.18	8.58E-05	0.24	1	0.97
rs7204325	16	23160007	SCNN1G	flanking_3UTR	0.21	8.81E-05	0.24	1	0.97
rs558692	2	161375373	RBMS1	flanking_5UTR	0.48	8.90E-05	-0.24	1	0.97
rs2341430	11	5546438	OR52B6	flanking_5UTR	0.06	9.01E-05	0.24	1	0.97
rs1384182	8	122575486	SNTB1	NA	0.16	9.16E-05	-0.24	1	0.97
rs1874094	4	164972837	40238	flanking_5UTR	0.38	9.25E-05	-0.24	1	0.97
rs5917876	23	39562048	BCOR	NA	0.36	9.25E-05	0.24	1	0.97
rs515279	1	4543548	AJAP1	NA	0.10	9.32E-05	0.24	1	0.97
rs4707640	6	91400378	MAP3K7	flanking_5UTR	0.38	9.34E-05	-0.24	1	0.97
rs928263	21	25433549	NA	NA	0.19	9.36E-05	-0.24	1	0.97
rs17734396	5	168374605	SLIT3	intron	0.28	9.45E-05	0.24	1	0.97
rs6755193	2	234366561	UGT1A3	flanking_3UTR	0.25	9.49E-05	-0.24	1	0.97
rs11654526	17	17558034	RAI1	intron	0.24	9.53E-05	0.24	1	0.97
rs17356990	8	123813965	ZHX2	flanking_5UTR	0.06	9.62E-05	0.24	1	0.97
rs4595159	8	17589144	MTUS1	intron	0.08	9.69E-05	0.24	1	0.97
rs1083694	6	69941856	BAI3	intron	0.20	9.70E-05	0.24	1	0.97
rs4707633	6	91384464	MAP3K7	NA	0.41	9.71E-05	-0.24	1	0.97
rs9324962	5	143941649	PRELID2	NA	0.22	9.96E-05	-0.24	1	0.97

Supplementary Table S5. **Integrated analysis for Rapamycin (SNPs vs. AUC, $P<10^{-4}$; SNPs vs. mRNA expression, $P<10^{-4}$; mRNA expression vs. AUC, $P<10^{-3}$).**

RAP	20 SNP-Gene Pairs													
**SNP ID**	**Chr**	**Closest Gene**	**SNP Location**	**Probe ID**	**Chr**	**Gene Symbol**	**SNP vs AUC**			**EXP vs AUC**		**SNP vs EXP**		
							**R**	**P**	**MAF**	**R**	**P**	**R**	**P**	**MAF**
rs2239104	12	CACNA1C	intron	212050_at	17	WIPF2	0.26	2.01E-05	0.28	0.23	9.98E-05	0.26	1.60E-05	0.28
rs2239104	12	CACNA1C	intron	214736_s_at	4	ADD1	0.26	2.01E-05	0.28	0.23	1.22E-04	0.24	6.62E-05	0.28
rs2239105	12	CACNA1C	intron	212050_at	17	WIPF2	0.27	1.68E-05	0.31	0.23	9.98E-05	0.26	1.36E-05	0.31
rs2239105	12	CACNA1C	intron	214736_s_at	4	ADD1	0.27	1.68E-05	0.31	0.23	1.22E-04	0.28	4.06E-06	0.31
rs10780752	9	C9orf153	unknown	222445_at	14	SLC39A9	-0.25	4.90E-05	0.31	0.24	6.79E-05	-0.26	2.03E-05	0.31
rs10780752	9	C9orf153	unknown	226560_at	2	---	-0.25	4.90E-05	0.31	0.21	3.72E-04	-0.25	5.10E-05	0.31
rs10780752	9	C9orf153	unknown	202377_at	1	---	-0.25	4.90E-05	0.31	0.21	6.71E-04	-0.26	1.37E-05	0.31
rs10918620	1	GPA33	intron	226577_at	14	PSEN1	0.25	4.18E-05	0.16	0.22	3.49E-04	0.26	2.25E-05	0.16
rs10918618	1	GPA33	intron	226577_at	14	PSEN1	0.25	4.91E-05	0.16	0.22	3.49E-04	0.26	1.75E-05	0.16
rs4144048	2	GYPC	flanking_5UTR	241155_at	10	PIP4K2A	0.25	6.20E-05	0.05	0.21	3.67E-04	0.26	1.13E-05	0.05
rs4144048	2	GYPC	flanking_5UTR	235790_at	14	LOC100131081	0.25	6.20E-05	0.05	-0.22	3.55E-04	-0.24	6.37E-05	0.05
rs2219206	2	GYPC	unknown	241155_at	10	---	0.27	1.56E-05	0.05	0.21	3.67E-04	0.25	3.75E-05	0.05
rs7543260	1	JUN	flanking_5UTR	203881_s_at	23	DMD	-0.24	9.78E-05	0.18	0.20	9.53E-04	-0.24	6.09E-05	0.18
rs10870177	9	MAN1B1	intron	218470_at	12	YARS2	-0.24	8.04E-05	0.16	-0.23	1.72E-04	0.24	5.26E-05	0.17
rs570046	18	ONECUT2	flanking_3UTR	201721_s_at	1	LAPTM5	-0.24	9.99E-05	0.44	0.23	1.47E-04	-0.28	4.13E-06	0.43
rs570046	18	ONECUT2	flanking_3UTR	201236_s_at	1	BTG2	-0.24	9.99E-05	0.44	0.27	6.97E-06	-0.25	3.65E-05	0.43
rs10515734	5	SGCD	flanking_5UTR	202932_at	18	YES1	-0.25	5.67E-05	0.12	0.25	2.55E-05	-0.28	3.87E-06	0.12
rs12655476	5	SGCD	unknown	202932_at	18	YES1	-0.25	7.29E-05	0.12	0.25	2.55E-05	-0.27	9.17E-06	0.12
rs588849	23	TMEM31	unknown	242750_at	4	MMAA	0.24	8.76E-05	0.18	0.22	2.57E-04	0.24	4.96E-05	0.18
rs588849	23	TMEM31	unknown	203906_at	3	IQSEC1	0.24	8.76E-05	0.18	0.25	3.70E-05	0.24	8.04E-05	0.18

Supplementary Table S6. Integrated analysis for Everolimus (SNPs vs. AUC, $P<10^{-4}$; SNPs vs. mRNA expression, $P<10^{-4}$; mRNA expression vs. AUC, $P<10^{-3}$).

EVE	20 SNP-Gene Pairs													
SNP ID	Chr	Closest Gene	SNP Location	Probe ID	Chr	Gene Symbol	SNP vs AUC			EXP vs AUC		SNP vs EXP		
							R	P	MAF	R	P	R	P	MAF
rs515279	1	AJAP1	unknown	201606_s_at	12	PWP1	0.24	9.32E-05	0.10	-0.20	9.13E-04	-0.24	6.19E-05	0.10
rs515279	1	AJAP1	unknown	1558688_at	9	LOC441461	0.24	9.32E-05	0.10	0.28	2.56E-06	0.25	4.65E-05	0.10
rs515279	1	AJAP1	unknown	1554646_at	18	OSBPL1A	0.24	9.32E-05	0.10	0.29	1.06E-06	0.25	4.08E-05	0.10
rs2832270	21	C21orf7	flanking_3UTR	1559007_s_at	5	---	0.28	8.39E-06	0.14	-0.20	8.14E-04	-0.25	2.78E-05	0.14
rs10780752	9	C9orf15	unknown	222445_at	14	SLC39A9	-0.26	3.49E-05	0.31	0.26	1.48E-05	-0.26	2.03E-05	0.31
rs17138777	10	C1QL3	unknown	1554952_s_at	19	NLRP12	0.25	6.73E-05	0.10	0.21	3.56E-04	0.28	2.92E-06	0.10
rs834063	7	DGKI	intron	200746_s_at	1	GNB1	-0.25	7.77E-05	0.10	0.20	8.10E-04	-0.24	6.06E-05	0.11
rs7786803	7	EMID2	flanking_3UTR	232271_at	8	HNF4G	0.26	1.98E-05	0.17	0.23	1.72E-04	0.26	2.37E-05	0.17
rs7543260	1	JUN	flanking_5UTR	203881_s_at	23	DMD	-0.26	2.35E-05	0.18	0.22	2.98E-04	-0.24	6.09E-05	0.18
rs6029138	20	MAFB	flanking_3UTR	229939_at	17	---	0.26	3.59E-05	0.19	0.25	3.50E-05	0.25	3.46E-05	0.19
rs10870177	9	MAN1B1	intron	218470_at	12	YARS2	-0.26	1.99E-05	0.16	-0.24	5.87E-05	0.24	5.26E-05	0.17
rs17732246	15	MCTP2	unknown	222792_s_at	12	CCDC59	-0.26	4.63E-05	0.10	-0.20	9.77E-04	0.24	7.74E-05	0.10
rs4595159	8	MTUS1	intron	228097_at	6	MYLIP	0.24	9.69E-05	0.08	-0.23	1.03E-04	-0.24	9.01E-05	0.08
rs4595159	8	MTUS1	intron	205635_at	3	KALRN	0.24	9.69E-05	0.08	0.23	1.45E-04	0.24	9.13E-05	0.08
rs6951882	7	NXPH1	flanking_3UTR	202942_at	19	ETFB	-0.26	3.30E-05	0.13	-0.22	1.93E-04	0.28	3.67E-06	0.13
rs6951882	7	NXPH1	flanking_3UTR	231276_at	11	PDE3B	-0.26	3.30E-05	0.13	-0.22	2.63E-04	0.24	7.42E-05	0.13
rs13272072	8	STC1	flanking_3UTR	224007_at	24	HSFY1 /// HSFY2	0.27	9.87E-06	0.10	0.23	1.54E-04	0.25	3.50E-05	0.11
rs11734813	4	SHROOM3	intron	216687_x_at	4	UGT2B15	0.26	2.04E-05	0.07	0.20	7.32E-04	0.24	9.77E-05	0.07
rs12932018	16	USP10	coding-synon	222368_at	8	---	0.28	5.78E-06	0.25	0.20	9.13E-04	0.24	9.85E-05	0.25
rs17356990	8	ZHX2	flanking_5UTR	224007_at	24	HSFY1 /// HSFY2	0.24	9.62E-05	0.06	0.23	1.54E-04	0.29	1.32E-06	0.06

Supplementary Table S7. miR-10a vs. mRNA expression association.

miRNA vs. mRNA							
mRNA			miR-10a vs. mRNA Exp			mRNA Exp vs. AUC	
Probe.id	Gene Symbol	Chr.	P	R	pred_ binding	P	R
229147_at	RASSF6	4	7.89E-14	0.48	0	0.0000689	0.24
**204082_at**	**PBX3**	**9**	**7.13E-12**	**0.48**	**0**	**0.00000345**	**0.28**
**217996_at**	**PHLDA1**	**12**	**2.26E-11**	**0.43**	**1**	**0.0000448**	**0.24**
221796_at	NTRK2	9	2.83E-11	0.44	1	0.0000871	0.23
204730_at	RIMS3	1	3.04E-11	0.43	1	0.0000519	0.24
202932_at	YES1	18	5.95E-10	0.42	1	0.0000255	0.25
238513_at	PRRG4	11	2.26E-09	0.39	0	0.0000155	0.26
**229419_at**	**FBXW7**	**4**	**5.36E-09**	**0.37**	**1**	**0.0000195**	**0.25**
228980_at	RFFL	17	7.52E-09	0.38	0	0.0000189	0.25
**222729_at**	**FBXW7**	**4**	**2.22E-08**	**0.37**	**1**	**0.0000478**	**0.24**
217788_s_at	GALNT2	1	2.57E-08	0.38	1	0.0000794	0.24
214783_s_at	ANXA11	10	4.00E-08	0.36	0	0.0000246	0.25
**228071_at**	**GIMAP7**	**7**	**1.98E-07**	**-0.38**	**0**	**0.0000391**	**-0.25**
209105_at	NCOA1	2	7.65E-07	0.36	1	0.0000452	0.24
1555062_s_at	GTPBP3	19	8.04E-07	-0.3	0	0.0000584	-0.24
238567_at	SGPP2	2	8.79E-07	0.34	0	0.0000686	0.24
226034_at	DUSP4	8	9.92E-07	0.32	0	0.0000996	0.23
**1552315_at**	**GIMAP1**	**7**	**1.54E-06**	**-0.34**	**0**	**0.0000963**	**-0.23**
**228355_s_at**	**NDUFAF2**	**5**	**2.13E-06**	**-0.31**	**0**	**0.0000475**	**-0.25**
205504_at	BTK	X	2.54E-06	-0.32	1	0.0000189	-0.25
227402_s_at	UTP23	8	5.29E-06	-0.31	1	0.0000583	-0.24
1560225_at	CNR1	6	6.69E-06	0.29	1	0.0000209	0.25
203906_at	IQSEC1	3	8.23E-06	0.31	0	0.000037	0.25
210001_s_at	SOCS1	16	8.61E-06	0.3	0	0.0000839	0.24
**229367_s_at**	**GIMAP6**	**7**	**1.54E-05**	**-0.31**	**1**	**0.00000979**	**-0.26**
**225102_at**	**MGLL**	**3**	**3.47E-05**	**0.28**	**1**	**0.0000394**	**0.25**
222997_s_at	MRPS21	1	4.22E-05	-0.27	0	0.0000673	-0.24
203907_s_at	IQSEC1	3	5.43E-05	0.29	0	0.0000582	0.24
202531_at	IRF1	5	6.22E-05	0.29	0	0.0000218	0.25
**1552316_a_at**	**GIMAP1**	**7**	**6.57E-05**	**-0.3**	**0**	**0.0000816**	**-0.24**
**207320_x_at**	**STAU1**	**20**	**8.90E-05**	**0.27**	**1**	**0.0000248**	**0.25**

predict binding sites for miR-10a and on the target genes.
Bold indicates genes that were followed up functionally.
